# Calibrating fit-index cutoffs for sparse ordinal CFA: Scenario-specific thresholds and operating characteristics

**DOI:** 10.3758/s13428-026-03221-8

**Published:** 2026-11-04

**Authors:** Liu Liu, Jin Liu, David Okech

**Affiliations:** 1 School of Social Work, University of Georgia, Athens, GA USA; 2 College of Education, University of Texas at Arlington, Arlington, TX USA

**Keywords:** Sparse ordinal data, Confirmatory factor analysis, Category collapsing, Scenario-specific fit-index cutoffs, Composite decision rules

## Abstract

Sparse ordinal response patterns can shift the sampling distributions of common confirmatory factor analysis (CFA) fit indices, making fixed heuristic cutoffs difficult to interpret across design conditions. We conducted a Monte Carlo simulation to calibrate scenario-specific cutoffs for the comparative fit index (CFI), Tucker–Lewis index (TLI), root mean square error of approximation (RMSEA), and standardized root mean square residual (SRMR) in sparse five-category ordinal CFA and to evaluate their operating characteristics. Data were generated from a three-factor population model with one cross-loading. For each dataset, we fitted a Correct model, a Local Misfit model omitting the cross-loading, and a Severe Misfit model combining two factors. We varied estimator (robust maximum likelihood [MLR], mean- and variance-adjusted maximum likelihood [MLMV], weighted least squares mean- and variance-adjusted maximum likelihood [WLSMV]), sample size (*N* = 250, 500, 1,000), loading strength (λ =  .50 vs. .80), sparsity pattern, sparse-item scope, and category handling (original vs. collapsed). Cutoffs were calibrated from Correct-model distributions at α =  .05, using lower-tail quantiles for CFI and TLI and upper-tail quantiles for RMSEA and SRMR. Calibrated thresholds were strongly scenario-dependent. RMSEA and SRMR cutoffs were generally far below conventional benchmarks, whereas CFI and TLI cutoffs varied with sample size and loading strength, falling below .95 in lower-information settings and approaching boundary values in larger-sample and high-loading settings. Detection was substantially lower for Local Misfit than for Severe Misfit. A calibrated CFI-RMSEA AND rule provided a transparent multi-index operating profile, with false-rejection and detection trade-offs that varied across scenarios. Collapsing produced modest changes for robust ML estimators but increased WLSMV detection in lower-information settings. We provide cutoff tables, operating-characteristic summaries, an interpolation aid, and an applied illustration to support scenario-calibrated fit evaluation in sparse ordinal CFA.

**Supplementary Information:**

The online version contains supplementary material available at https://doi.org/10.3758/s13428-026-03221-8.

## Introduction

Goodness-of-fit (GOF) indices are central to evaluating model fit in confirmatory factor analysis (CFA) and structural equation modeling (SEM) (Jackson et al., 2009; Schermelleh-Engel et al., 2003). Researchers routinely report indices such as the comparative fit index (CFI; Bentler, 1990), Tucker–Lewis index (TLI; Tucker & Lewis, 1973), root mean square error of approximation (RMSEA; Steiger, 1990), and standardized root mean square residual (SRMR), often alongside chi-square-based tests (Hu & Bentler, 1999; Schermelleh-Engel et al., 2003). These quantities serve complementary roles in model evaluation. The chi-square test evaluates the null hypothesis of exact model fit and provides a formal significance test, whereas descriptive fit indices summarize different aspects of approximate, relative, or residual fit. CFI and TLI evaluate improvement relative to a baseline model, RMSEA summarizes discrepancy per degree of freedom, and SRMR summarizes standardized residual discrepancies. Both forms of fit information are also design-dependent. The chi-square test is sensitive to sample size and can detect small discrepancies in large samples, while descriptive fit indices may also vary with sample size, loading magnitude, model complexity, estimator, and distributional characteristics even when those features are not themselves forms of model misspecification (Garrido et al., 2016; Heene et al., 2011; Moshagen, 2012; Moshagen & Auerswald, 2018; Ximénez et al., 2022). Accordingly, chi-square tests and descriptive fit indices provide complementary information about model fit (Zheng & Bentler, 2025).

The best-known heuristics trace to Hu and Bentler (1999), who proposed widely used benchmarks such as CFI and TLI around .95 or higher, RMSEA near .06 or lower, and SRMR near .08 or lower. These cutoffs remain widely used as default interpretive rules in applied CFA and SEM (Jackson et al., 2009; Marsh et al., 2004). However, a large body of methodological literature shows that GOF indices reflect not only model misspecification but also design and analysis features that vary across applications, including sample size, loading magnitude and corresponding residual variance, model complexity, estimator choice, response distribution, and indicator scaling (Chen et al., 2008; Garrido et al., 2016; Heene et al., 2011; Kenny & McCoach, 2003; Moshagen, 2012; Moshagen & Auerswald, 2018; Shi et al., 2019; Ximénez et al., 2022). As a result, a fixed cutoff can have different empirical operating characteristics across settings. In one scenario, a cutoff may rarely flag a correctly specified model; in another, the same cutoff may flag a correctly specified model much more often. This context dependence has motivated growing interest in fit-index evaluation strategies that make the reference scenario explicit.

### Tailored cutoffs and scenario-specific decision rules

Several approaches have been proposed to tailor fit evaluation to the modeling context (Bollen & Stine, 1992; MacCallum et al., 1996; McNeish, 2023; Nye & Drasgow, 2011). Some strategies derive fit decisions from chi-square-based reference distributions or equivalence-style tests of close fit (MacCallum et al., 1996; Moshagen & Erdfelder, 2016). Others use meta-analytic simulations and regression equations to predict scenario-dependent cutoffs from study characteristics (Groskurth et al., 2024; Nye & Drasgow, 2011). A third strategy is scenario-specific simulation-based calibration, in which researchers generate reference distributions of fit indices under a target model, sample size, response structure, and estimator (Groskurth et al., 2024; Millsap, 2007, 2013; Niemand & Mai, 2018; Pornprasertmanit, 2014).

The scenario-specific approach is especially useful when the empirical setting differs from the conditions under which conventional cutoffs were originally studied. In this framework, fit indices are treated as approximate-fit summaries whose decision use can be characterized by simulated operating characteristics. Cutoffs can be obtained from the Correct-model reference distribution, for example by selecting the lower-tail α quantile for incremental indices such as CFI and TLI and the upper-tail 1 − α quantile for absolute misfit indices such as RMSEA and SRMR (Groskurth et al., 2024; McNeish & Wolf, 2023). The resulting cutoff describes how extreme an observed index is relative to the Correct-model reference distribution for that scenario. Prespecified misspecified models can then be used to evaluate detection, defined as the probability that the same cutoff or decision rule flags a model with a known departure from the data-generating structure. This approach yields an empirical profile of fit-index behavior under both correct specification and targeted forms of misspecification.

Recent work has also emphasized population-targeted and bias-corrected perspectives on fit assessment, including unbiased or population-oriented forms of residual fit indices such as SRMR (Maydeu-Olivares, 2017; Shi et al., 2020; Ximénez et al., 2022). These approaches are especially relevant when sample-based fit measures reflect finite-sample behavior, estimator-specific scaling, or distributional features in addition to substantive model misfit. Conventional software-reported CFI, TLI, RMSEA, and SRMR remain widely used in cutoff-based model evaluation in applied SEM, making their behavior under sparse ordinal conditions practically important.

### Sparse ordinal CFA as a motivating applied context

Sparse ordinal CFA is a common and practically important context in which fixed GOF cutoffs are especially likely to have scenario-dependent operating characteristics. Likert-type indicators are ubiquitous in psychological and social science measurement, and in many applications some response options are rarely endorsed. Sparse extreme categories may occur in symptom scales, stigma or victimization measures, risk-behavior items, household hardship indicators, and other instruments in which the most severe or least severe categories are uncommon. Such response patterns produce sparse marginal distributions and sparse bivariate contingency tables, both of which are consequential for ordinal CFA (DiStefano et al., 2021; Forero et al., 2009; Rhemtulla et al., 2012).

Ordinal CFA is commonly estimated using either limited-information categorical estimators based on polychoric correlations, such as weighted least squares mean- and variance-adjusted (WLSMV), or robust maximum-likelihood variants that treat ordinal indicators as approximately continuous, such as robust maximum likelihood (MLR) and mean- and variance-adjusted ML (MLMV) (Flora & Curran, 2004; Forero et al., 2009; Rhemtulla et al., 2012; Savalei, 2012). These estimators differ not only in how they represent ordinal responses but also in how their model test statistics are adjusted. MLR retains maximum-likelihood parameter estimation and uses sandwich-based robust standard errors together with a chi-square test statistic that is asymptotically equivalent to the Yuan–Bentler ${T}_{2}^{*}$ test statistic. MLMV also uses maximum-likelihood parameter estimation but employs a mean- and variance-adjusted chi-square test statistic that is robust to non-normality. In contrast, WLSMV treats the indicators as ordered categorical variables and estimates the model using a diagonal weight matrix, with standard errors and a mean- and variance-adjusted chi-square test statistic based on the full weight matrix (Muthén & Muthén, 1998–2017). Together, these estimators contrast two robust ML test-statistic adjustments with a categorical weighted least-squares framework. These estimation frameworks also provide different pathways through which sparse ordinal response patterns can affect global fit indices. Under MLR and MLMV, ordinal responses are treated as approximately continuous, so sparsity can influence the observed moments, distributional non-normality, and the estimator-specific robust chi-square adjustment. Under WLSMV, sparse response patterns can additionally affect estimated thresholds, polychoric correlations, and the weight-matrix information used in estimation and test-statistic adjustment.

Sparsity is often discussed as an estimation issue, particularly for WLSMV-type methods that rely on adequate bivariate cell counts for polychoric estimation. When categories are empty or very rare, polychoric estimation can become unstable, and downstream CFA estimation can exhibit nonconvergence, improper solutions, or unstable standard errors (Flora & Curran, 2004; Forero et al., 2009; Savalei, 2012). A common applied response is to collapse adjacent categories to increase effective cell counts, which can improve stability and estimation quality under severe sparsity (DiStefano et al., 2021). Robust ML approaches are sometimes used as an alternative when sparsity is limited or items have several response categories, although performance can change under severe threshold asymmetry, skew, or strong departures from the continuous-variable approximation (Rhemtulla et al., 2012).

Sparse ordinal response patterns also affect the behavior of fit indices. For limited-information categorical estimators, sparse marginal categories create sparse bivariate contingency tables, and these tables provide the empirical basis for estimating thresholds, polychoric correlations, and their associated weight matrices. When extreme categories are empty or nearly empty, the estimated association structure can become more variable across samples even when the underlying factor model is correctly specified. This variability then propagates into the discrepancy functions, baseline-model comparisons, residual association summaries, and robust test-statistic adjustments from which global fit indices are computed. For robust ML estimators that treat ordinal indicators as approximately continuous, sparsity operates through a different pathway: highly asymmetric category use changes the observed mean, variance, and covariance structure of the discretized indicators, so the fitted continuous-variable approximation can yield fit-index distributions that differ from those expected under more balanced response scales. Collapsing categories modifies both pathways by increasing effective cell counts and reducing threshold extremity, while also changing the observed response scale and the information available for detecting localized misfit. These mechanisms make sparse ordinal CFA a setting in which fit-index cutoffs are expected to depend jointly on estimator, sample size, loading strength, sparsity pattern, and category handling.

### Current gaps

Several gaps motivate the present study. First, fit-index calibration and sparse-category handling have only recently been examined together. Dynamic and scenario-specific cutoff work for ordinal CFA has emphasized factors such as sample size, loading strength, model complexity, response distributions, and estimator choice (Groskurth et al., 2024; McNeish, 2023; Xia & Yang, 2019), whereas category-collapsing research has focused more heavily on estimation quality and model-fit behavior (DiStefano et al., 2021; J. Liu et al., 2026). Recent simulation-cum-ROC (receiver operating characteristic) evidence further shows that fit-index discrimination under sparse ordinal CFA can vary with category collapsing and information conditions (L. Liu et al., 2026). What remains less developed is the calibration of Correct-model reference distributions under these conditions and the false-rejection and detection profiles of cutoffs derived from them. A key applied need is therefore to translate these context-dependent shifts in GOF distributions into scenario-calibrated reference values that support fit-index interpretation under sparse-category conditions.

Second, sparse ordinal CFA is frequently estimated using different estimator families, including robust ML variants and WLSMV-type limited-information estimators (Flora & Curran, 2004; Forero et al., 2009; Rhemtulla et al., 2012). Estimator choice can shift GOF index values and their behavior, creating applied ambiguity in interpretation (Savalei, 2021; Xia & Yang, 2019). Under severe sparsity and collapsing, this ambiguity may be amplified because estimators use information differently in sparse contingency tables and because their discrepancy statistics can behave differently. Applied researchers therefore need scenario-calibrated evidence linking estimator family and sparse-category handling to the behavior of standard GOF indices.

Third, applied SEM practice typically considers multiple indices, but the behavior of simple multi-index decision rules remains underexplored under sparse ordinal conditions. Hu and Bentler (1999) proposed a two-index strategy, and subsequent work shows that multi-index rules can vary across designs and misspecification types (Fan & Sivo, 2005; Marsh et al., 2004). In sparse-category ordinal CFA, estimator choice, information loss, and collapsing decisions can all shift index behavior (Flora & Curran, 2004; Rhemtulla et al., 2012; Xia & Yang, 2019). This leaves an open question about how transparent conjunctive rules, such as pairing an incremental index with an absolute misfit index, and majority-style rules trade off false rejection under correct specification against detection under targeted misspecification. These rules provide simple extensions of the common practice of considering multiple fit indices jointly and can be implemented directly once scenario-calibrated component cutoffs are available.

### The present study

The present study extends scenario-specific simulation-based calibration to sparse ordinal CFA with category collapsing. We examine how the reference distributions of CFI, TLI, RMSEA, and SRMR vary across sparse ordinal response conditions and use those distributions to derive scenario-calibrated cutoffs with explicit operating characteristics. We then evaluate how these cutoffs balance false rejection under correct specification with detection under targeted forms of misspecification.

We operationalize sparsity using predefined response-pattern cells that vary in the number of items exhibiting sparse categories and in the severity of sparse category frequencies, including empty or near-empty extreme categories. We examine three frequentist estimators, MLR, MLMV, and WLSMV, to evaluate whether scenario-specific fit-index calibration depends on the robust test-statistic adjustment within maximum likelihood and on the broader distinction between robust ML and categorical weighted least-squares estimation.

Within each full simulation cell, we calibrate cutoffs for CFI, TLI, RMSEA, and SRMR from the Correct-model sampling distributions at α = .05, using lower-tail quantiles for CFI and TLI and upper-tail 1 − α quantiles for RMSEA and SRMR (Groskurth et al., 2024; McNeish & Wolf, 2023). We then summarize the cell-level cutoffs within each Estimator × Category handling × Sample size × Loading scenario and evaluate the operating characteristics of single-index and composite decisions under three fitted-model conditions. The Correct model retains the data-generating factor structure and is used to summarize false rejection under correct specification. The Local Misfit model omits a prespecified cross-loading while retaining the three-factor structure, providing a localized misspecification condition. The Severe Misfit model combines the first two factors into a two-factor structure, providing a stronger misspecification condition. The Local and Severe Misfit models are used to evaluate detection after cutoffs have been calibrated from the Correct model.

Because applied researchers often interpret multiple fit indices jointly, we also evaluate transparent composite rules that combine calibrated component cutoffs. We summarize their empirical operating characteristics directly, including false rejection under the Correct condition and detection under Local and Severe Misfit, allowing direct comparison of these trade-offs across scenarios. Finally, we include a worked applied illustration using a real sparse ordinal scale to demonstrate how researchers can document sparse response structure, compare original and collapsed category handling, fit an ordinal CFA model, and conduct a tailored mini-simulation to obtain a model-conditional reference distribution for the empirical setting. We also provide prediction equations as a rough interpolation tool for settings without a close simulation match (Groskurth et al., 2024; Nye & Drasgow, 2011).

More specifically, we address three research questions:

1. How do scenario-calibrated cutoffs for CFI, TLI, RMSEA, and SRMR vary across estimator family, sample size, loading strength, sparsity patterns, and category handling, and how do they compare with commonly used fixed benchmarks?
2. Using those cutoffs, how do single indices and transparent composite rules balance false rejection under correct specification with detection under Local and Severe Misfit, and how does this depend on estimator family and category handling?
3. Under what conditions does collapsing sparse categories meaningfully change calibrated cutoffs and the operating characteristics of single-index and composite decision rules, and how do these changes vary across estimator and information conditions?

To support applied use, we report scenario-level cutoff tables and operating characteristics, estimator-specific summaries, and a worked empirical illustration of the calibration workflow.

## Method

### Simulation design and data generation

We conducted a Monte Carlo simulation of CFA with sparse five-category ordinal indicators. The design calibrated scenario-specific cutoffs from Correct-model sampling distributions, evaluated false rejection and detection under Correct, Local Misfit, and Severe Misfit fitted-model conditions, and compared original with collapsed category handling.

**Fig. 1:**
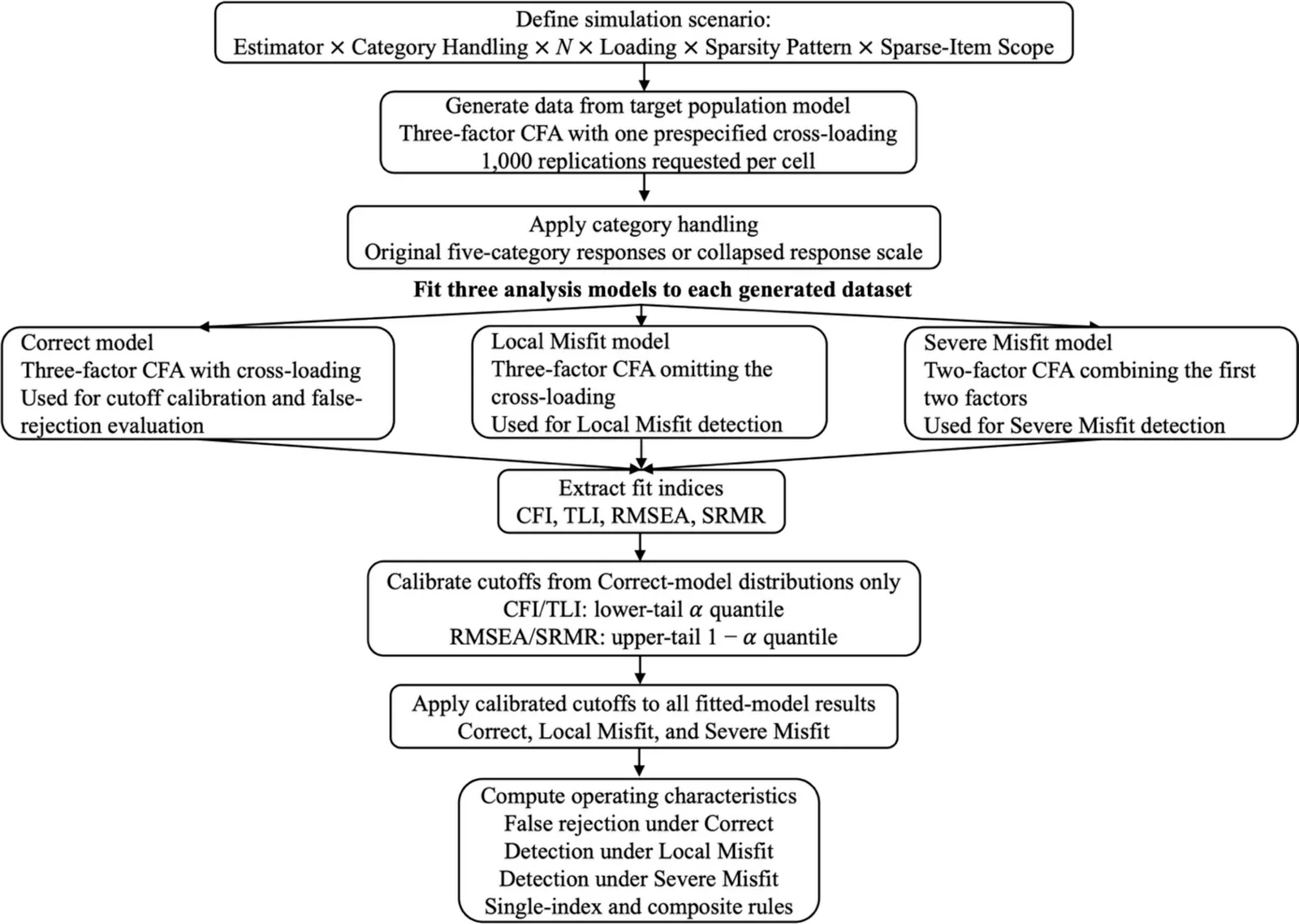
Workflow for scenario-specific cutoff calibration. *Note.* Simulation workflow and fitted-model conditions. Data were generated from the target three-factor population model with one prespecified cross-loading. Each generated dataset was analyzed using three fitted models: the Correct model, which retained the cross-loading; the Local Misfit model, which omitted the cross-loading while retaining the three-factor structure; and the Severe Misfit model, which combined the first two factors into a two-factor structure. Cutoffs were calibrated only from the Correct-model reference distributions and were then applied to the Correct, Local Misfit, and Severe Misfit results to estimate false rejection and detection for single-index and composite decision rules

Within each design cell, data were generated from the corresponding target population model. For each generated dataset, we fitted three analysis models: a Correct model, a Local Misfit model, and a Severe Misfit model. The Correct model provided the reference distributions used for cutoff calibration, whereas the Local and Severe Misfit models were used only to evaluate detection after cutoffs had been calibrated from the Correct model. Figure 1 summarizes the simulation workflow, fitted-model conditions, and cutoff-based evaluation scheme. The fitted-model conditions are described in detail in the next section.

The population model was a three-factor CFA in which each factor was measured primarily by five indicators, yielding 15 indicators in total. Factor 1 was measured primarily by ${u}_{1}$ through ${u}_{5}$, Factor 2 by ${u}_{6}$ through ${u}_{10}$, and Factor 3 by ${u}_{11}$ through ${u}_{15}$. Two loading regimes were examined. In the moderate-loading condition, all primary loadings were set to λ = .50, whereas in the high-loading condition, all primary loadings were set to λ = .80. Indicator ${u}_{6}$ had its primary loading on Factor 2 and an additional secondary loading of .30 on Factor 1. Thus, the loading vector for ${u}_{6}$ across Factors 1, 2, and 3 was ${\left(.30,.\mathrm{50,0}\right)}^{^{\prime}}$ in the moderate-loading condition and ${\left(.30,.\mathrm{80,0}\right)}^{^{\prime}}$ in the high-loading condition.

Factor variances were fixed at 1, and all pairwise factor correlations were fixed at .40. Accordingly, the factor covariance matrix was

$$\Phi =\left(\begin{array}{ccc}1& .40& .40\\ .40& 1& .40\\ .40& .40& 1\end{array}\right).$$

Residual variances were specified so that each indicator had unit variance on the underlying continuous response scale. For indicator ${u}_{6}$, the true-score variance was calculated as ${\lambda}_{{u}_{6}}^{^{\prime}}\Phi {\lambda}_{{u}_{6}}$. In the moderate-loading condition, this variance was.

${.30}^{2}+{.50}^{2}+2(.30)(.50)(.40)=.460,$ yielding a residual variance of 1-.460=.540. In the high-loading condition, the true-score variance was

$${.30}^{2}+{.80}^{2}+2(.30)(.80)(.40)=.922,$$

yielding a residual variance of 1-.922=.078. Because the Factor 3 loading was zero, its covariance terms were also zero. For indicators without a secondary loading, residual variances were set to $1-{\lambda }^{2}$.

Holding the target population structure fixed provided a common basis for examining how sample size, loading strength, response sparsity, category handling, and estimator affect fit-index behavior (DiStefano et al., 2021; Flora & Curran, 2004; Rhemtulla et al., 2012; Xia & Yang, 2019).

Observed responses were generated as five-category ordinal variables by discretizing the underlying continuous indicators using thresholds selected to reproduce target marginal category proportions. Sparsity was operationalized through predefined response-pattern cells that varied along two dimensions: sparse-item scope and sparsity severity. Sparse-item scope varied across 1, 2, or 4 items per factor. Sparsity severity was represented by three marginal distributions containing empty, near-empty, or low-frequency extreme categories in the original-category condition. Table 1 shows the target marginal category proportions and the deterministic collapsing rules used in the collapsed-category condition.

**Table 1:** Marginal category proportions used to induce sparsity and corresponding proportions after collapsing

Sparsity pattern	Percentages before collapsing (5 categories)	Percentages after collapsing
1 sparse category	22%, 40%, 20%, 16%, 2%	22%, 40%, 20%, 18%
2 sparse categories	22%, 50%, 22%, 4%, 2%	22%, 50%, 28%
4 sparse categories	88%, 4%, 4%, 2%, 2%	88%, 12%

These marginal category proportions define the sparse-response patterns used in the simulation. Collapsing merges extremely sparse categories with adjacent categories, reducing the number of response categories as shown. The collapsing rule is applied after data generation, prior to model estimation, in the collapsed-category conditions

The simulation crossed sample size (*N* = 250, 500, 1,000), loading regime (moderate vs. high), category handling (original vs. collapsed), estimator (MLR, MLMV, WLSMV), sparsity pattern (three marginal distributions), and sparse-item scope (1, 2, or 4 items per factor). This produced 324 full design cells for each fitted-model condition: 3 estimators × 2 category-handling conditions × 3 sample sizes × 2 loading regimes × 3 sparsity patterns × 3 sparse-item scopes. For each generated dataset in each cell, we fitted the Correct, Local Misfit, and Severe Misfit analysis models, resulting in 972 fitted model-by-design-cell combinations.

We requested 1,000 replications per design cell. Primary summaries were based on replications for which the corresponding fit-index value was available, with unavailable values omitted from the relevant index-specific summary. We additionally conducted a replication-level audit of nonconvergence, improper solutions, and unavailable fit indices across all fitted model-by-design-cell combinations. Improper-solution flags were based on Mplus warnings for inadmissible parameter solutions, including negative residual variances (Heywood cases) and non-positive-definite residual or latent covariance matrices. Some replications with improper-solution warnings still returned fit-index values. We therefore repeated the cutoff and operating-characteristic calculations after excluding these replications as a sensitivity analysis. Condition-specific replication diagnostics are reported in Supplementary Table S1.

### Fitted-model conditions

The Correct fitted model matched the target data-generating structure. It specified three correlated factors, five primary indicators per factor, the prespecified secondary loading of ${u}_{6}$ on Factor 1, and factor variances fixed for scale identification. The Correct model was used to estimate Correct-model fit-index reference distributions and to summarize fit-index behavior under correct specification.

The Local Misfit fitted model retained the three-factor structure but fixed the secondary loading of ${u}_{6}$ on Factor 1 to zero. This condition represented a localized misspecification of the measurement structure while preserving the broader factor organization and provided a targeted test of sensitivity to an omitted secondary loading.

The Severe Misfit fitted model combined the first two factors into a single factor while retaining the third factor as separate. This condition represented a stronger structural departure from the data-generating factor structure and provided a contrast with the localized omitted-cross-loading condition.

Only the Correct model contributed to cutoff calibration. The Local and Severe Misfit models were used only to evaluate detection after cutoffs had been calibrated from the Correct-model distributions.

### Category handling and estimation

Category handling was implemented as a paired analysis condition. For each sparse-data scenario, we analyzed the generated responses in two ways: retaining the original five-category response scale and applying the deterministic collapsing rules shown in Table 1 before model estimation. Original and collapsed analyses therefore shared the same underlying population model, sample size, loading regime, sparsity pattern, and sparse-item scope, but differed in how sparse response categories were represented during estimation.

Accordingly, collapsed-minus-original contrasts are interpreted as the net change associated with moving from the sparse original five-category response scale to the corresponding collapsed response scale. This comparison captures a common applied modeling decision in sparse ordinal CFA: whether to retain the original response categories or collapse low-frequency categories before evaluating model fit.

All models were estimated in Mplus 8.11 using estimator-default settings. We examined MLR, MLMV, and WLSMV to represent two distinct estimation approaches and, within the robust ML approach, two different test-statistic adjustments. For MLR and MLMV, the generated ordinal responses were analyzed as approximately continuous variables. MLR used maximum-likelihood estimation with sandwich-based robust standard errors and a chi-square test statistic asymptotically equivalent to the Yuan–Bentler ${T}_{2}^{*}$ statistic, whereas MLMV used maximum-likelihood estimation with robust standard errors and a mean- and variance-adjusted chi-square test statistic. For WLSMV, the indicators were specified as ordered categorical using the CATEGORICAL option; parameter estimation used a diagonal weight matrix, with standard errors and the mean- and variance-adjusted test statistic based on the full weight matrix (Muthén & Muthén, 1998–2017). This comparison distinguishes differences between the two robust ML test-statistic adjustments from the broader contrast between robust continuous-variable and categorical-data estimation.

### Fit-index definitions

For each fitted model, we extracted four global fit indices as reported by Mplus: CFI, TLI, RMSEA, and SRMR. These indices were selected because they are widely reported in applied CFA and SEM and are commonly interpreted using cutoff-based decision rules. To make their relationships with the underlying model-fit statistics explicit, let ${\upchi}_{M}^{2}$ and ${df}_{M}$ denote the estimator-specific chi-square test statistic and degrees of freedom for the fitted target model, and let ${\upchi}_{B}^{2}$ and ${df}_{B}$ denote the corresponding quantities for the baseline model. Let *N* denote the sample size.

CFI was defined as

$$\mathrm{C}\mathrm{F}\mathrm{I}=1-\frac{\mathrm{max}\left({\upchi}_{M}^{2} - {df}_{M}, 0\right)}{\mathrm{max}\left({\upchi}_{M}^{2} - {df}_{M}, {\upchi}_{B }^{2}- {df}_{B}, 0\right)}.$$

TLI was defined as

$$\mathrm{T}\mathrm{L}\mathrm{I}=\frac{{\upchi}_{B}^{2}/{df}_{B} - {\upchi}_{M}^{2}/{df}_{M}}{{\upchi}_{B}^{2}/{df}_{B} - 1}.$$

For the single-group models considered here, RMSEA was defined as

$$\mathrm{R}\mathrm{M}\mathrm{S}\mathrm{E}\mathrm{A}=\sqrt{max(\frac{{\upchi}_{M}^{2}-{df}_{M}}{{Ndf}_{M}}, 0)}.$$

These expressions make clear that CFI, TLI, and RMSEA are functions of the chi-square test statistic. CFI and TLI additionally incorporate the chi-square statistic and degrees of freedom from the baseline model. Accordingly, estimator-specific adjustments to the target-model and baseline-model chi-square statistics can propagate to CFI, TLI, and RMSEA. For MLR, MLMV, and WLSMV, the quantities ${\upchi}_{M}^{2}$ and ${\upchi}_{B}^{2}$ therefore refer to the corresponding estimator-specific robust or adjusted test statistics reported by Mplus (Muthén & Muthén, 1998–2017).

In contrast, SRMR is based directly on standardized residual discrepancies between observed and model-implied quantities rather than on the chi-square test statistic. Its general form can be expressed as

$$\mathrm{S}\mathrm{R}\mathrm{M}\mathrm{R}=\sqrt{\frac{1}{q}\sum_{k=1}^{q}{r}_{k}^{2}},$$

where ${r}_{k}$ denotes a standardized residual element and *q* denotes the number of residual elements included in the calculation. Thus, unlike CFI, TLI, and RMSEA, SRMR is not directly affected by the estimator-specific adjustment applied to the chi-square test statistic. The precise residual elements entering SRMR depend on the model and outcome type as implemented in Mplus. We report TLI in addition to CFI because the two indices use the same target- and baseline-model fit information but differ in their treatment of model degrees of freedom and complexity, allowing us to assess whether conclusions generalize across closely related incremental fit indices.

### Scenario-specific cutoff calibration

The cutoff-calibration analysis focused on CFI, TLI, RMSEA, and SRMR because these descriptive fit indices are commonly interpreted using heuristic benchmarks whose operating characteristics may vary across design conditions. The chi-square test serves a complementary role as a formal test of exact fit and is reported alongside the descriptive fit indices in the applied illustration. Scenario-specific cutoffs were calibrated from the Correct-model sampling distribution of each fit index, consistent with simulation-based calibration approaches that define tailored thresholds as tail quantiles of the Correct-model reference distribution (Groskurth et al., 2024; McNeish & Wolf, 2023; Millsap, 2007, 2013; Niemand & Mai, 2018; Pornprasertmanit, 2014). Cutoffs were first computed at the full-cell level, defined by Estimator × Category handling × Sample size × Loading regime × Sparsity pattern × Sparse-item scope.

For incremental indices, where larger values indicate better fit (CFI and TLI), the index-specific cutoff was defined as the lower-tail α quantile of the Correct-model reference distribution. At the primary calibration level of α = .05, this corresponded to the 5th percentile, and a replication was flagged as misfitting when the index value fell below the cutoff. For absolute misfit indices, where larger values indicate worse fit (RMSEA and SRMR), the cutoff was defined as the upper-tail 1 − α quantile of the Correct-model reference distribution. At α = .05, this corresponded to the 95th percentile, and a replication was flagged as misfitting when the index value exceeded the cutoff. Here, α denotes the tail probability used to define the calibrated reference threshold. We used α = .05 for the primary analyses and repeated the calibration at α = .01 and α = .10 as sensitivity checks.

### Operating characteristics and decision rules

To support applied interpretation, we summarized cell-level results at the broader scenario level. A scenario was defined by Estimator × Category handling × Sample size × Loading regime. Each scenario contained nine sparsity cells, formed by crossing three sparsity patterns with three sparse-item scopes. Cutoffs were first calibrated within each full cell. Scenario-level cutoffs were then obtained by taking the unweighted mean of the corresponding cell-level cutoffs across the nine sparsity cells. The unweighted mean was used so that each prespecified sparsity configuration contributed equally to the broader scenario summary. We also recorded the minimum and maximum cell-level cutoffs within each scenario to summarize within-scenario variability across sparsity conditions.

Operating characteristics were summarized at the scenario level using the scenario-level cutoffs. For each scenario, false rejection was defined as the proportion of Correct-model replications flagged as misfitting. Local Misfit detection was defined as the proportion of Local Misfit replications flagged as misfitting. Severe Misfit detection was defined as the proportion of Severe Misfit replications flagged as misfitting. These rates were computed by applying the scenario-level cutoff to replications with the fit index or indices required for the corresponding decision available, pooled across the nine sparsity cells within each scenario. Because each sparsity cell was designed to contribute the same number of requested replications, the pooled summaries provide an interpretable scenario-level operating profile while retaining the empirical replication counts available for each condition.

After calibration, we evaluated single-index decisions for CFI, TLI, RMSEA, and SRMR. A replication was flagged as misfitting when an index fell on the worse-fit side of its scenario-specific cutoff, with direction defined by the index. For CFI and TLI, values below the cutoff were flagged as misfitting. For RMSEA and SRMR, values above the cutoff were flagged as misfitting.

We also evaluated three transparent composite decision rules. Following the two-index strategy that pairs an incremental fit index with an absolute misfit index, we evaluated a conjunctive CFI-RMSEA AND rule. Fit was judged acceptable when both CFI and RMSEA met their calibrated thresholds, that is, when CFI was at or above its cutoff and RMSEA was at or below its cutoff. We also evaluated a 2-of-3 rule based on CFI, RMSEA, and SRMR. Fit was judged acceptable when at least two of the three indices met their calibrated thresholds. We also evaluated a CFI-RMSEA OR acceptance rule. Fit was judged acceptable when either CFI or RMSEA met its calibrated threshold. These rules were selected a priori as transparent multi-index decision structures that can be implemented directly from calibrated component cutoffs.

Composite rules were evaluated as decision rules with their own empirical operating characteristics. Single-index cutoffs were calibrated from the corresponding Correct-model marginal distributions and then combined within each rule. Because the component indices are jointly distributed, the overall false-rejection rate of a composite rule is an empirical property of that joint distribution. We therefore estimated composite-rule false rejection directly under the Correct condition and summarized its variation across scenarios. These summaries characterize the trade-off between false rejection under correct specification and detection under Local and Severe Misfit for each rule.

To quantify Monte Carlo uncertainty in the operating-characteristic estimates, we computed approximate 95% Monte Carlo confidence intervals for false rejection and detection rates. For each estimated rate, p, the Monte Carlo standard error was computed as $\sqrt{p(1-p)/R}$, where *R* is the number of replications with the required fit-index value available for the corresponding estimate. Confidence intervals were computed as p ± 1.96 × MCSE. These intervals were used to summarize the precision of scenario-level false-rejection and detection estimates in the main and supplemental tables.

### Prediction equations and sensitivity checks

To provide an interpolation tool when an exact scenario match is unavailable, we fitted quantile regression models to the scenario-level cutoffs calibrated at α = .05. Scenario-level cutoffs were defined as the unweighted mean across the nine sparsity cells within each Estimator × Category handling × Sample size × Loading regime scenario. Separate models were fitted for each index, using τ = .05 for CFI and TLI and τ = .95 for RMSEA and SRMR. Predictors included log_10_(*N*), loading regime, category-handling indicator, and estimator contrasts with WLSMV as the reference category. Bootstrap standard errors were used for coefficient inference.

Approximation accuracy was summarized using in-sample absolute errors and leave-one-scenario-out cross-validation, where each held-out unit was one Estimator × Category handling × Sample size × Loading regime scenario. We used log_10_(*N*) to obtain a parsimonious interpolation function. As a robustness check, we refit the models treating *N* as a three-level categorical predictor, with results reported in the supplement.

We conducted several sensitivity and robustness checks. First, we recalibrated cutoffs at α = .01 and α = .10 to examine sensitivity to the calibration level. Second, we trimmed the most extreme sparsity tier and recomputed cutoffs and operating characteristics to assess the influence of the most severe sparse-category conditions. Third, we repeated the cutoff and operating-characteristic calculations after excluding replications flagged as improper to assess sensitivity to the admissibility screen. In addition, we quantified Monte Carlo uncertainty for operating-characteristic estimates using approximate 95% Monte Carlo confidence intervals based on the replication count contributing to each estimate. Finally, we summarized realized false rejection for single-index and composite rules under the Correct condition, including deviations from the nominal .05 reference level, to characterize the empirical error profile of cutoff-based decision rules.

## Results

### Scenario-specific cutoffs for CFI, TLI, RMSEA, and SRMR

Across fitted model-by-design-cell combinations, the number of replications with available fit-index results ranged from 819 to 1,000. The replication-level audit identified 55 nonconvergence flags and 3,998 improper-solution flags; these diagnostic categories were not necessarily mutually exclusive. Detailed nonconvergence, improper-solution, and available-replication counts are reported in Supplementary Table S1. We also repeated the cutoff and operating-characteristic analyses after excluding improper solutions. For the focal CFI-RMSEA AND rule, the maximum absolute changes were .002 for false rejection, .009 for Local Misfit detection, and .020 for Severe Misfit detection.

**Fig. 2:**
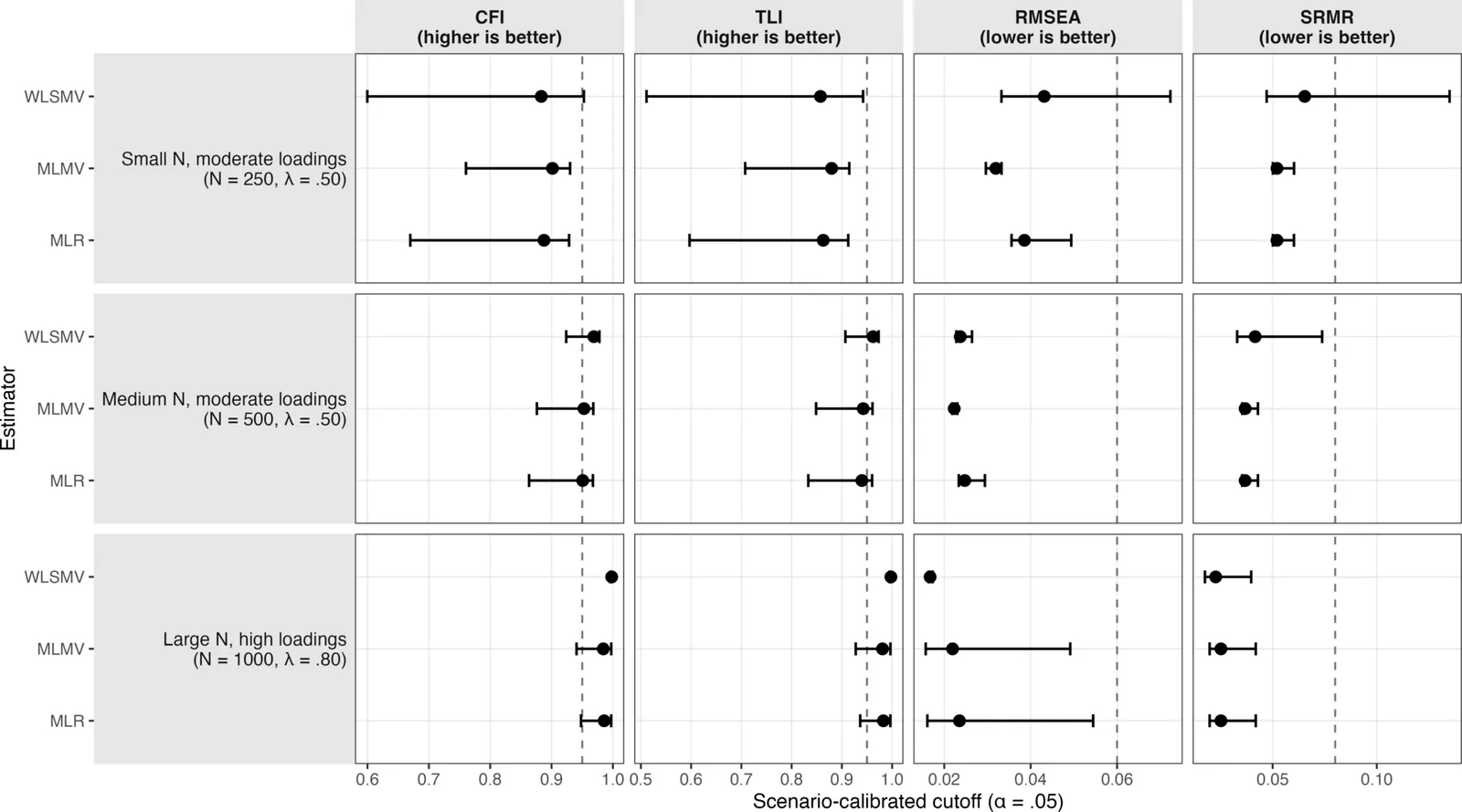
Scenario-specific calibrated cutoffs under correct specification for incremental (CFI, TLI) and absolute (RMSEA, SRMR) fit indices in selected sparse ordinal CFA scenarios. *Note.* The displayed scenarios were selected to illustrate lower-, intermediate-, and higher-information conditions. Points show scenario-level cutoffs calibrated at α = .05 from the Correct-model reference distributions. Error bars show the minimum and maximum cell-level cutoffs across sparsity cells within each scenario, not confidence intervals. Dashed vertical lines mark conventional heuristic benchmarks: CFI/TLI = .95, RMSEA = .06, and SRMR = .08. The *x*-axis scale varies across indices. For CFI and TLI, higher values indicate better fit; for RMSEA and SRMR, lower values indicate better fit

**Fig. 3:**
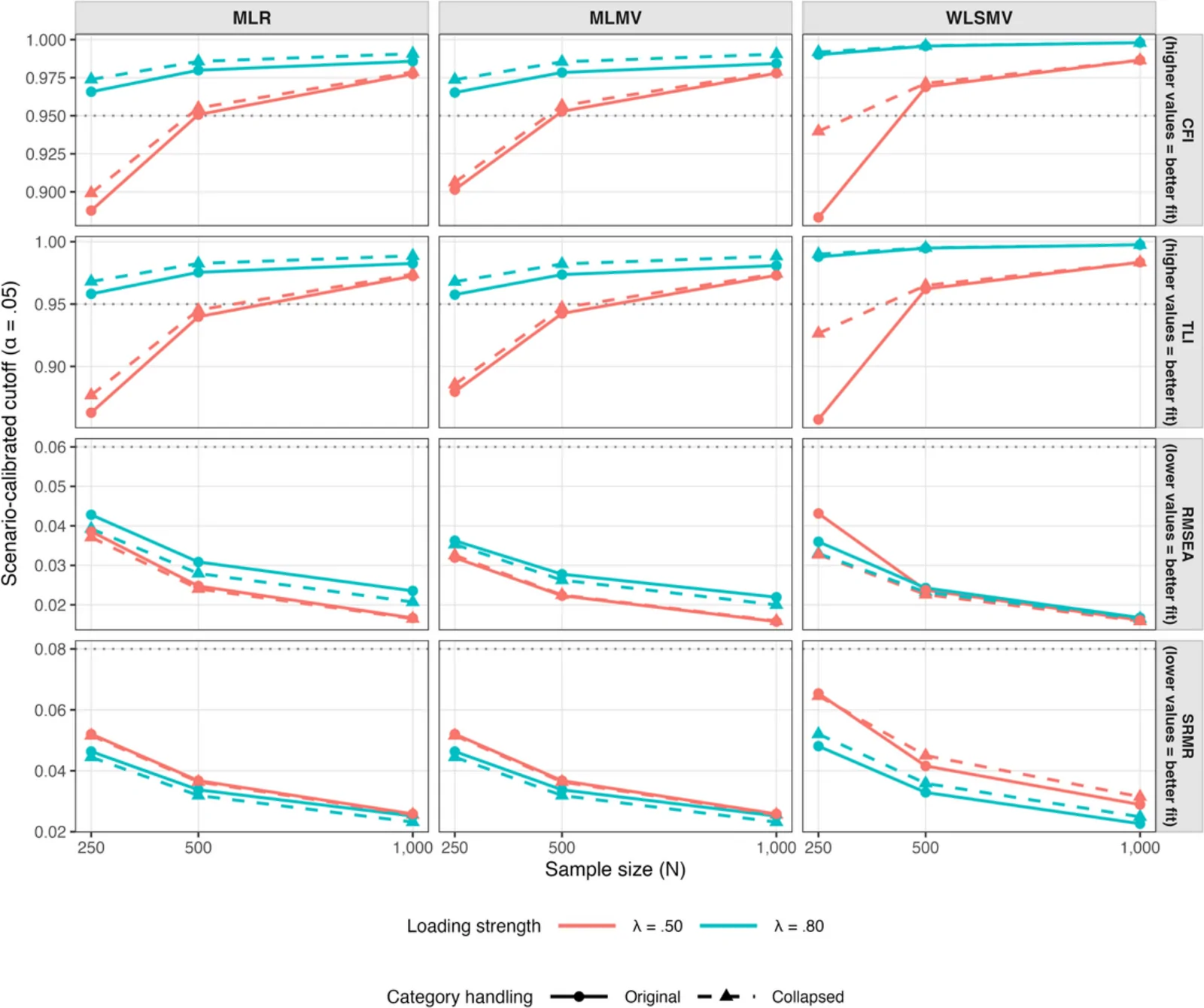
Scenario-calibrated Correct-model cutoffs by sample size, loading strength, estimator, and category handling. *Note.* Points show scenario-level cutoffs calibrated at α = .05 from the Correct-model reference distributions. Color denotes loading strength (λ = .50 vs. λ = .80). Solid lines with circles represent the original five-category response scale, whereas dashed lines with triangles represent the paired collapsed response scale. Dotted horizontal lines mark conventional heuristic benchmarks: CFI/TLI = .95, RMSEA = .06, and SRMR = .08. The *y*-axis scale varies across fit indices. For CFI and TLI, higher values indicate better fit; for RMSEA and SRMR, lower values indicate better fit. Original and collapsed analyses were paired within the same sparse-data scenarios, so differences between the corresponding lines summarize the change associated with moving from the sparse original response scale to the collapsed response scale

Table 2 reports scenario-calibrated cutoffs for CFI, TLI, RMSEA, and SRMR across estimators, sample sizes, loading strengths, and category-handling conditions. Cutoffs were calibrated at α = .05 from the Correct-model sampling distributions. For CFI and TLI, cutoffs were defined as the lower-tail 5th percentile. For RMSEA and SRMR, cutoffs were defined as the upper-tail 95th percentile. Figures 2 and 3 visualize these calibrated cutoffs alongside conventional heuristic reference values, CFI/TLI = .95, RMSEA = .06, and SRMR = .08. Additional estimator-specific cutoff displays are provided in Fig. S1. Across scenarios, the calibrated cutoffs often diverged from the conventional reference values, showing that the empirical reference distribution for each fit index changed with estimator, sample size, loading strength, category handling, and sparsity configuration.

**Table 2:** Scenario-calibrated Correct-model cutoffs for CFI, TLI, RMSEA, and SRMR at α = .05

Estimator	Category handling	*N*	Loading	CFI cutoff	TLI cutoff	RMSEA cutoff	SRMR cutoff
MLR	Original	250	High	.9657 [.8809, .9863]	.9582 [.8546, .9832]	.0428 [.0349, .0704]	.0463 [.0398, .0683]
		250	Moderate	.8877 [.6698, .9285]	.8629 [.5968, .9127]	.0386 [.0356, .0494]	.0521 [.0501, .0602]
		500	High	.9799 [.9316, .9935]	.9755 [.9165, .9921]	.0309 [.0242, .0594]	.0338 [.0280, .0518]
		500	Moderate	.9507 [.8634, .9674]	.9399 [.8332, .9602]	.0248 [.0234, .0294]	.0368 [.0354, .0429]
		1,000	High	.9858 [.9480, .9971]	.9826 [.9365, .9965]	.0235 [.0161, .0545]	.0252 [.0197, .0419]
		1,000	Moderate	.9775 [.9391, .9854]	.9725 [.9256, .9822]	.0167 [.0158, .0189]	.0258 [.0247, .0303]
	Collapsed	250	High	.9739 [.9170, .9860]	.9681 [.8987, .9829]	.0393 [.0351, .0524]	.0445 [.0397, .0601]
		250	Moderate	.8992 [.7604, .9256]	.8769 [.7074, .9092]	.0371 [.0349, .0422]	.0516 [.0504, .0571]
		500	High	.9857 [.9526, .9936]	.9825 [.9421, .9921]	.0279 [.0240, .0432]	.0319 [.0282, .0445]
		500	Moderate	.9551 [.8924, .9683]	.9452 [.8686, .9612]	.0240 [.0233, .0265]	.0364 [.0352, .0406]
		1,000	High	.9907 [.9651, .9970]	.9887 [.9574, .9964]	.0207 [.0161, .0384]	.0232 [.0198, .0344]
		1,000	Moderate	.9787 [.9498, .9853]	.9740 [.9387, .9821]	.0165 [.0159, .0173]	.0256 [.0248, .0287]
MLMV	Original	250	High	.9652 [.8865, .9863]	.9576 [.8614, .9833]	.0362 [.0321, .0565]	.0463 [.0398, .0683]
		250	Moderate	.9015 [.7605, .9302]	.8797 [.7076, .9147]	.0319 [.0296, .0333]	.0521 [.0501, .0603]
		500	High	.9784 [.9266, .9937]	.9736 [.9104, .9923]	.0278 [.0231, .0515]	.0338 [.0280, .0518]
		500	Moderate	.9529 [.8761, .9680]	.9424 [.8487, .9610]	.0223 [.0218, .0230]	.0368 [.0354, .0429]
		1,000	High	.9843 [.9407, .9972]	.9808 [.9276, .9966]	.0219 [.0157, .0492]	.0252 [.0197, .0419]
		1,000	Moderate	.9779 [.9409, .9855]	.9731 [.9278, .9824]	.0158 [.0153, .0161]	.0258 [.0247, .0303]
	Collapsed	250	High	.9737 [.9183, .9862]	.9679 [.9002, .9832]	.0353 [.0324, .0471]	.0445 [.0397, .0601]
		250	Moderate	.9063 [.8003, .9274]	.8856 [.7562, .9113]	.0325 [.0312, .0335]	.0516 [.0504, .0571]
		500	High	.9854 [.9508, .9937]	.9822 [.9399, .9924]	.0263 [.0228, .0408]	.0319 [.0282, .0445]
		500	Moderate	.9566 [.9001, .9688]	.9470 [.8781, .9619]	.0225 [.0222, .0230]	.0364 [.0352, .0406]
		1,000	High	.9904 [.9634, .9971]	.9883 [.9554, .9965]	.0200 [.0157, .0370]	.0232 [.0198, .0344]
		1,000	Moderate	.9790 [.9504, .9855]	.9744 [.9394, .9823]	.0159 [.0155, .0166]	.0256 [.0248, .0287]
WLSMV	Original	250	High	.9901 [.9721, .9936]	.9879 [.9660, .9922]	.0360 [.0337, .0433]	.0481 [.0355, .0919]
		250	Moderate	.8832 [.5997, .9527]	.8574 [.5113, .9422]	.0431 [.0332, .0723]	.0654 [.0471, .1350]
		500	High	.9958 [.9909, .9969]	.9948 [.9889, .9962]	.0243 [.0232, .0251]	.0329 [.0250, .0595]
		500	Moderate	.9690 [.9239, .9780]	.9621 [.9070, .9731]	.0237 [.0228, .0264]	.0416 [.0329, .0738]
		1,000	High	.9980 [.9959, .9985]	.9976 [.9950, .9981]	.0167 [.0164, .0173]	.0226 [.0175, .0397]
		1,000	Moderate	.9865 [.9701, .9901]	.9835 [.9634, .9879]	.0161 [.0156, .0169]	.0289 [.0230, .0504]
	Collapsed	250	High	.9918 [.9835, .9937]	.9900 [.9799, .9923]	.0330 [.0313, .0337]	.0521 [.0359, .1048]
		250	Moderate	.9398 [.8795, .9539]	.9265 [.8529, .9437]	.0327 [.0311, .0340]	.0646 [.0478, .1240]
		500	High	.9960 [.9922, .9968]	.9951 [.9905, .9961]	.0232 [.0217, .0250]	.0359 [.0253, .0704]
		500	Moderate	.9712 [.9365, .9785]	.9649 [.9225, .9738]	.0226 [.0220, .0231]	.0450 [.0334, .0858]
		1,000	High	.9980 [.9957, .9984]	.9975 [.9948, .9980]	.0165 [.0160, .0168]	.0249 [.0177, .0482]
		1,000	Moderate	.9865 [.9704, .9900]	.9835 [.9638, .9878]	.0159 [.0154, .0164]	.0315 [.0235, .0594]

Values are scenario-level cutoffs calibrated from the Correct-model reference distributions at α = .05. For CFI and TLI, cutoffs correspond to the lower-tail 5th percentile, and values below the cutoff indicate poorer fit relative to the Correct-model reference distribution. For RMSEA and SRMR, cutoffs correspond to the upper-tail 95th percentile, and values above the cutoff indicate poorer fit relative to the Correct-model reference distribution. Bracketed values are the minimum and maximum cell-level cutoffs across the nine sparsity cells within each Estimator × Category handling × Sample size × Loading scenario, reported as [min, max]. Original indicates that the sparse five-category response scale was retained. Collapsed indicates that sparse categories were collapsed according to the deterministic recoding rules in Table 1 before model estimation. CFI = comparative fit index; TLI = Tucker–Lewis index; RMSEA = root mean square error of approximation; SRMR = standardized root mean square residual; MLR = robust maximum likelihood; MLMV = mean and variance adjusted maximum likelihood; WLSMV = weighted least squares mean and variance adjusted

Because each Estimator × Category handling × *N* × Loading scenario contains multiple sparsity cells, Table 2 reports scenario-level summaries. The point estimate is the mean cutoff across the underlying sparsity cells defined by sparsity pattern and sparse-item scope. Bracketed values report the corresponding minimum and maximum cell-level cutoffs within each scenario. These ranges show how much the calibrated cutoff varied as the severity of sparsity and the number of affected items changed within the same broader scenario. Full cell-level cutoffs are available as a supplementary CSV file in the Open Science Framework repository (https://osf.io/ce7rm/) to support closer matching to specific sparsity configurations.

A central pattern revealed that calibrated RMSEA cutoffs were well below the conventional .06 benchmark across estimators and scenarios. At α = .05, scenario-level RMSEA cutoffs ranged from approximately .04 in the small-sample, moderate-loading settings to approximately .016 in larger-sample settings. With original categories and moderate loadings, RMSEA cutoffs decreased with *N* for robust ML estimators. For MLR, the cutoff decreased from .0386 at *N* = 250 to .0248 at *N* = 500 and .0167 at *N* = 1,000. For MLMV, the corresponding cutoffs decreased from .0319 to .0223 and .0158. Accordingly, the calibrated Correct-model upper-tail reference for RMSEA was well below .06 in these sparse ordinal CFA scenarios.

CFI and TLI cutoffs varied strongly with loading strength and sample size. Under high loadings, calibrated incremental-index cutoffs exceeded .95 even at *N* = 250 and increased as *N* increased. With original categories, the CFI cutoff was .9657 for MLR and .9652 for MLMV at *N* = 250, increasing to .9858 for MLR and .9843 for MLMV at *N* = 1,000. WLSMV yielded still higher CFI cutoffs in the same high-loading original-category scenarios, increasing from .9901 at *N* = 250 to .9980 at *N* = 1,000. Under moderate loadings at *N* = 250, robust ML cutoffs were lower than .95 in the original-category condition—for example, .8877 for MLR and .9015 for MLMV. TLI followed the same general pattern and was typically slightly lower than CFI within the same scenario. The conventional .95 reference point therefore occupied different positions relative to the Correct-model distributions across scenarios.

SRMR cutoffs were also smaller than the conventional .08 benchmark. At α = .05, scenario-level SRMR cutoffs were typically between approximately .02 and .07, depending on *N*, loading strength, estimator, and category handling. Thus, the conventional SRMR benchmark of .08 was generally higher than the calibrated Correct-model upper-tail reference in the sparse ordinal settings examined here.

Together, Table 2 and Figs. 2 and 3 show that sparse ordinal CFA produces scenario-dependent fit-index reference distributions. RMSEA and SRMR calibrated cutoffs were consistently below conventional benchmarks. CFI and TLI cutoffs occupied different positions relative to .95 depending on *N* and loading strength, falling below .95 in small-sample, moderate-loading settings and rising above .95 in large-sample or high-loading settings. Sensitivity tables for α = .01 and α = .10 showed corresponding shifts in the cutoffs, with similar directional patterns across *N*, loading strength, estimator, and category handling.

These cutoff patterns are consistent with the information structure of sparse ordinal CFA. In lower-information settings, such as smaller *N*, moderate loadings, and sparse original categories, fit-index distributions were more dispersed and calibrated cutoffs moved farther from conventional benchmarks. In higher-information settings, such as larger *N* and high loadings, incremental-index distributions were concentrated closer to their upper boundary, producing higher CFI and TLI cutoffs. Consistent with the estimator-specific pathways described earlier, the observed estimator differences indicate that sparse category information can affect fit-index behavior differently under robust ML estimation and WLSMV.

### Operating characteristics of single indices and composite rules

**Fig. 4:**
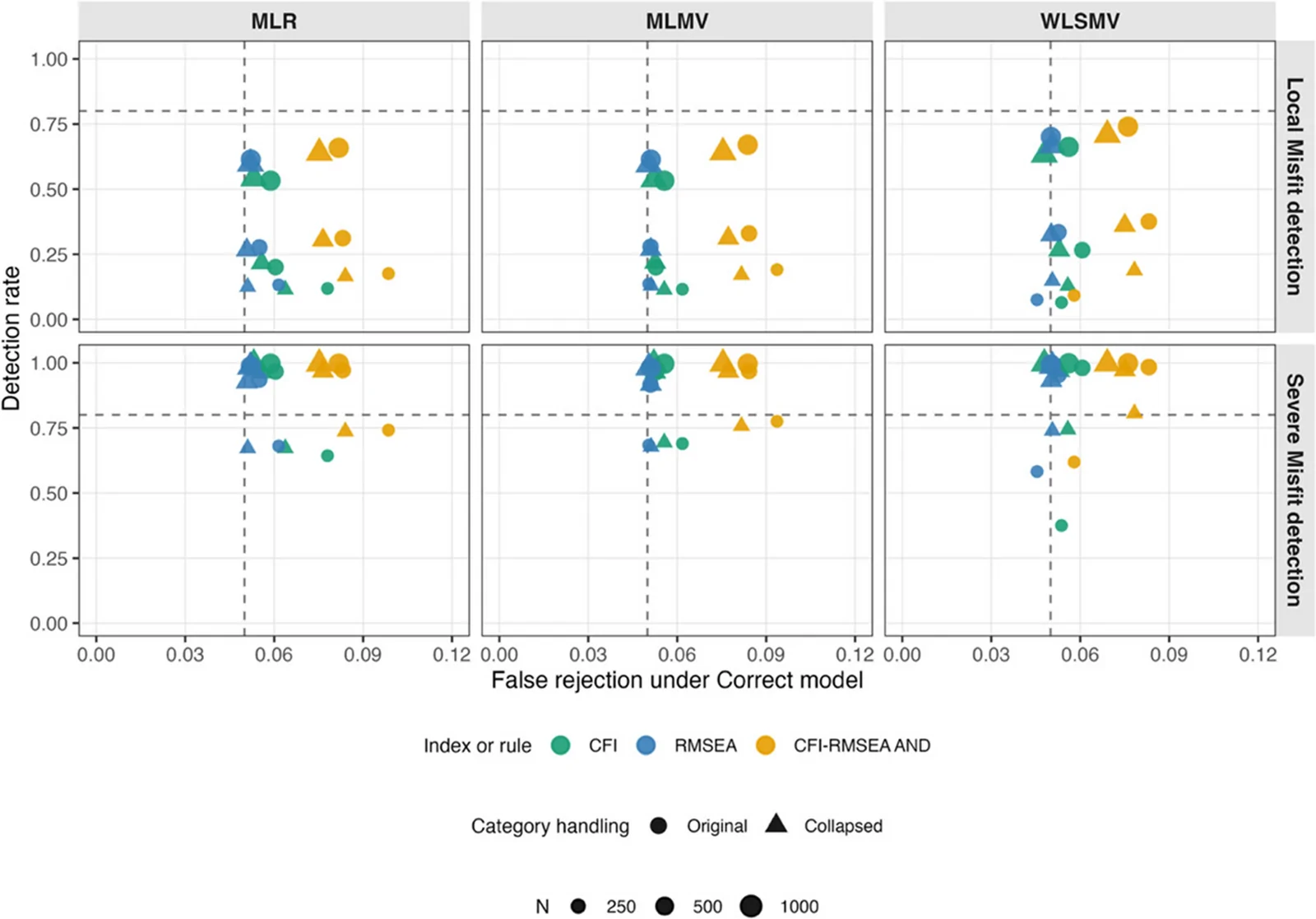
Operating characteristics for core fit-index decisions under moderate loadings. *Note.* The *x*-axis shows false rejection under the Correct fitted model, and the *y*-axis shows detection under the Local Misfit fitted model, which omitted the prespecified cross-loading, or the Severe Misfit fitted model, which combined the first two factors. Points show scenario-level results for CFI, RMSEA, and the CFI-RMSEA AND rule across estimator, category handling, and sample size. Dashed vertical lines mark the .05 false-rejection reference level, and dashed horizontal lines mark .80 detection as a descriptive visual reference. These lines were not used to define or calibrate the decision rules. Cutoffs were calibrated from Correct-model reference distributions before being applied to Correct, Local Misfit, and Severe Misfit results. CFI = comparative fit index; RMSEA = root mean square error of approximation

Table 3 summarizes operating characteristics under moderate loadings for the core fit-index decisions emphasized in the main text: CFI, RMSEA, and the CFI-RMSEA AND rule. Values are reported as false rejection under the Correct model, detection under the Local Misfit model, and detection under the Severe Misfit model, with approximate 95% Monte Carlo confidence intervals. The full grid of operating characteristics across estimators, category-handling conditions, sample sizes, loading regimes, single indices, and composite rules is reported in Table S2. Figure 4 complements the tables by plotting scenario-level operating characteristics for the core decisions under Local and Severe Misfit, enabling a visual comparison of the trade-off between false rejection under correct specification and detection under misspecification.

**Table 3:** Operating characteristics for core fit-index decisions under moderate loadings using scenario-calibrated cutoffs

Estimator	Category handling	*N*	Rule	False rejection	Local Misfit detection	Severe Misfit detection
MLR	Original	250	CFI	0.078 [0.072, 0.084]	0.119 [0.112, 0.125]	0.644 [0.634, 0.654]
		250	RMSEA	0.062 [0.057, 0.066]	0.132 [0.125, 0.139]	0.681 [0.671, 0.690]
		250	CFI-RMSEA AND	0.099 [0.092, 0.105]	0.176 [0.168, 0.184]	0.742 [0.733, 0.751]
		500	CFI	0.060 [0.056, 0.065]	0.201 [0.192, 0.209]	0.966 [0.963, 0.970]
		500	RMSEA	0.055 [0.050, 0.060]	0.276 [0.267, 0.286]	0.936 [0.931, 0.941]
		500	CFI-RMSEA AND	0.083 [0.077, 0.089]	0.312 [0.302, 0.321]	0.972 [0.969, 0.976]
		1,000	CFI	0.059 [0.054, 0.064]	0.533 [0.522, 0.543]	0.997 [0.995, 0.998]
		1,000	RMSEA	0.052 [0.048, 0.057]	0.614 [0.604, 0.624]	0.986 [0.984, 0.989]
		1,000	CFI-RMSEA AND	0.082 [0.076, 0.087]	0.658 [0.649, 0.668]	0.997 [0.995, 0.998]
	Collapsed	250	CFI	0.064 [0.059, 0.069]	0.115 [0.108, 0.122]	0.672 [0.662, 0.682]
		250	RMSEA	0.051 [0.047, 0.056]	0.125 [0.118, 0.132]	0.672 [0.662, 0.682]
		250	CFI-RMSEA AND	0.084 [0.078, 0.090]	0.165 [0.158, 0.173]	0.737 [0.728, 0.746]
		500	CFI	0.056 [0.051, 0.061]	0.217 [0.208, 0.225]	0.965 [0.961, 0.969]
		500	RMSEA	0.051 [0.046, 0.055]	0.266 [0.257, 0.275]	0.927 [0.922, 0.932]
		500	CFI-RMSEA AND	0.076 [0.071, 0.082]	0.304 [0.295, 0.314]	0.968 [0.964, 0.971]
		1,000	CFI	0.053 [0.048, 0.058]	0.541 [0.531, 0.551]	0.997 [0.995, 0.998]
		1,000	RMSEA	0.052 [0.047, 0.057]	0.598 [0.588, 0.608]	0.985 [0.982, 0.988]
		1,000	CFI-RMSEA AND	0.075 [0.070, 0.081]	0.639 [0.629, 0.649]	0.997 [0.995, 0.998]
MLMV	Original	250	CFI	0.062 [0.057, 0.067]	0.116 [0.109, 0.122]	0.690 [0.681, 0.700]
		250	RMSEA	0.050 [0.046, 0.055]	0.136 [0.129, 0.143]	0.684 [0.675, 0.694]
		250	CFI-RMSEA AND	0.094 [0.088, 0.100]	0.191 [0.183, 0.199]	0.775 [0.766, 0.784]
		500	CFI	0.053 [0.048, 0.057]	0.200 [0.192, 0.208]	0.963 [0.959, 0.967]
		500	RMSEA	0.051 [0.046, 0.056]	0.279 [0.270, 0.288]	0.916 [0.911, 0.922]
		500	CFI-RMSEA AND	0.084 [0.078, 0.090]	0.330 [0.320, 0.340]	0.969 [0.965, 0.972]
		1,000	CFI	0.056 [0.051, 0.060]	0.533 [0.522, 0.543]	0.996 [0.995, 0.998]
		1,000	RMSEA	0.051 [0.047, 0.056]	0.614 [0.603, 0.624]	0.981 [0.978, 0.984]
		1,000	CFI-RMSEA AND	0.084 [0.078, 0.090]	0.671 [0.661, 0.680]	0.996 [0.995, 0.998]
	Collapsed	250	CFI	0.056 [0.051, 0.060]	0.114 [0.108, 0.121]	0.695 [0.685, 0.704]
		250	RMSEA	0.051 [0.047, 0.056]	0.130 [0.123, 0.137]	0.678 [0.669, 0.688]
		250	CFI-RMSEA AND	0.082 [0.076, 0.087]	0.171 [0.163, 0.179]	0.758 [0.749, 0.766]
		500	CFI	0.053 [0.048, 0.057]	0.219 [0.210, 0.228]	0.964 [0.960, 0.968]
		500	RMSEA	0.051 [0.047, 0.056]	0.268 [0.259, 0.277]	0.918 [0.912, 0.923]
		500	CFI-RMSEA AND	0.077 [0.072, 0.083]	0.312 [0.302, 0.321]	0.967 [0.964, 0.971]
		1,000	CFI	0.052 [0.048, 0.057]	0.537 [0.527, 0.548]	0.997 [0.995, 0.998]
		1,000	RMSEA	0.050 [0.046, 0.055]	0.594 [0.584, 0.604]	0.982 [0.979, 0.985]
		1,000	CFI-RMSEA AND	0.075 [0.070, 0.081]	0.641 [0.631, 0.651]	0.997 [0.995, 0.998]
WLSMV	Original	250	CFI	0.054 [0.049, 0.058]	0.065 [0.060, 0.070]	0.375 [0.365, 0.386]
		250	RMSEA	0.045 [0.041, 0.050]	0.075 [0.070, 0.081]	0.582 [0.572, 0.593]
		250	CFI-RMSEA AND	0.058 [0.053, 0.063]	0.092 [0.086, 0.099]	0.619 [0.609, 0.629]
		500	CFI	0.061 [0.056, 0.066]	0.266 [0.257, 0.275]	0.981 [0.978, 0.984]
		500	RMSEA	0.053 [0.048, 0.057]	0.335 [0.325, 0.345]	0.954 [0.949, 0.958]
		500	CFI-RMSEA AND	0.083 [0.077, 0.089]	0.376 [0.366, 0.386]	0.984 [0.981, 0.986]
		1,000	CFI	0.056 [0.051, 0.061]	0.662 [0.652, 0.672]	0.998 [0.997, 0.999]
		1,000	RMSEA	0.050 [0.046, 0.055]	0.700 [0.691, 0.710]	0.993 [0.992, 0.995]
		1,000	CFI-RMSEA AND	0.076 [0.071, 0.082]	0.740 [0.731, 0.749]	0.998 [0.997, 0.999]
	Collapsed	250	CFI	0.056 [0.051, 0.061]	0.129 [0.122, 0.136]	0.744 [0.735, 0.753]
		250	RMSEA	0.051 [0.046, 0.055]	0.149 [0.141, 0.156]	0.740 [0.731, 0.749]
		250	CFI-RMSEA AND	0.078 [0.073, 0.084]	0.188 [0.180, 0.196]	0.806 [0.798, 0.814]
		500	CFI	0.053 [0.048, 0.058]	0.266 [0.257, 0.275]	0.970 [0.966, 0.973]
		500	RMSEA	0.050 [0.046, 0.055]	0.325 [0.315, 0.334]	0.932 [0.927, 0.938]
		500	CFI-RMSEA AND	0.075 [0.070, 0.080]	0.361 [0.351, 0.370]	0.973 [0.970, 0.976]
		1,000	CFI	0.048 [0.043, 0.052]	0.632 [0.622, 0.642]	0.997 [0.996, 0.998]
		1,000	RMSEA	0.051 [0.046, 0.055]	0.668 [0.658, 0.678]	0.988 [0.986, 0.990]
		1,000	CFI-RMSEA AND	0.069 [0.064, 0.074]	0.708 [0.699, 0.718]	0.997 [0.996, 0.998]

Local Misfit refers to the omitted-cross-loading model. Severe Misfit refers to the two-factor model that combines the first two factors. Values are rates, with approximate 95% Monte Carlo confidence intervals in brackets. Composite rules use marginally calibrated cutoffs for their component indices. Composite-rule false-rejection rates are empirical operating characteristics of the joint rule under the Correct condition

Detection was consistently lower for the Local Misfit condition than for the Severe Misfit condition. The Local Misfit model retained the three-factor structure but omitted the prespecified cross-loading, whereas the Severe Misfit model combined the first two factors into a two-factor structure. The Local Misfit results therefore provide a targeted assessment of sensitivity to localized measurement misspecification, while the Severe Misfit results summarize detection under a stronger structural departure.

Under moderate loadings, the CFI-RMSEA AND rule produced a mean false-rejection rate of .080 across estimator, category-handling, and sample-size scenarios. Mean detection was .391 for Local Misfit and .903 for Severe Misfit. The median detection rate was .321 for Local Misfit and .970 for Severe Misfit, with Local Misfit detection ranging from .092 to .740 and Severe Misfit detection ranging from .619 to .998. These results show that the same calibrated rule can have very different sensitivity depending on whether the misspecification is localized or severe. Detection increased strongly with sample size, especially for the Local Misfit condition.

### Single-index decisions

#### CFI

Under moderate loadings, CFI detection increased with sample size for both Local and Severe Misfit. For robust ML estimators, Local Misfit detection was modest at *N* = 250, increased at *N* = 500, and became substantially higher by *N* = 1,000. For example, under MLR with original categories, Local Misfit detection increased from .119 at *N* = 250 to.201 at *N* = 500 and .533 at *N* = 1,000. Severe Misfit detection was much higher in the same scenarios, increasing from .644 to .966 and .997. WLSMV showed the lowest CFI detection in the original-category, *N* = 250, moderate-loading setting, with Local Misfit detection of .065 and Severe Misfit detection of .375. With collapsed categories in the same WLSMV setting, Local Misfit detection increased to .129 and Severe Misfit detection increased to .744. Under moderate loadings, CFI detection therefore varied substantially with sample size and category handling, with the largest differences occurring for WLSMV in small-sample sparse settings.

#### RMSEA

RMSEA showed stable false rejection near the nominal reference in most moderate-loading scenarios and increasing detection as *N* increased. For robust ML estimators, Local Misfit detection increased gradually with sample size, whereas Severe Misfit detection was already high by *N* = 500 and near ceiling by *N* = 1,000. For example, under MLR with original categories, Local Misfit detection increased from .132 at *N* = 250 to .276 at *N* = 500 and .614 at *N* = 1,000, while Severe Misfit detection increased from .681 to .936 and .986. Under WLSMV with original categories, RMSEA detection in the *N* = 250 moderate-loading condition was .075 for Local Misfit and .582 for Severe Misfit. With collapsed categories, the corresponding values increased to .149 and .740. RMSEA detection increased with sample size and differed substantially between Local and Severe Misfit across the moderate-loading scenarios.

#### SRMR

SRMR showed a distinct operating-characteristic profile. Across the full scenario grid, SRMR had higher false rejection than the other single-index decisions, with mean false rejection of .169, median false rejection of .199, and a range from .074 to .234. The pattern was most pronounced for WLSMV, where SRMR false rejection was elevated across several moderate-loading scenarios. In the *N* = 250 WLSMV original-category setting, SRMR combined elevated false rejection with comparatively low detection relative to CFI and RMSEA. Overall, SRMR’s decision-rule performance was less stable across the sparse ordinal conditions examined here, particularly for WLSMV.

### Composite decision rules

Composite rules were used to summarize the joint information provided by multiple fit indices and were evaluated through their empirical operating characteristics. Under moderate loadings, the CFI-RMSEA AND rule demonstrated a mean false-rejection rate of .080 across estimator, category-handling, and sample-size scenarios, with a median of .080 and a range from .058 to .099.

Across the full scenario grid, including both moderate- and high-loading conditions, the CFI-RMSEA AND rule showed broader variation in false rejection. Its mean false-rejection rate was .113, with a median of .083 and a maximum of .225. The largest values occurred primarily in high-loading robust-ML scenarios, where CFI and RMSEA were especially sensitive to small discrepancies and where scenario-level aggregation across sparsity cells contributed to higher realized false rejection. These results provide an explicit empirical error profile for the AND rule and help characterize the trade-off between false rejection under correct specification and detection under Local and Severe Misfit.

The conjunctive CFI-RMSEA AND rule often yielded higher detection than either component index alone. The difference was most visible in settings where one component index had comparatively low detection. For example, under WLSMV with original categories, *N* = 250, and moderate loadings, CFI alone achieved Local Misfit detection of .065 and Severe Misfit detection of .375. The CFI-RMSEA AND rule increased these values to .092 and .619 while maintaining a similar false-rejection rate. With collapsed categories in the same WLSMV setting, the AND rule increased Local Misfit detection to .188 and Severe Misfit detection to .806. As *N* increased, Severe Misfit detection approached 1.00 in many scenarios, while Local Misfit detection also increased across estimators.

The 2-of-3 rule based on CFI, RMSEA, and SRMR provided a majority-style alternative. For MLR and MLMV, its operating characteristics were often similar to the AND rule once *N* was at least 500. In the WLSMV, *N* = 250, moderate-loading scenarios, SRMR showed higher false rejection and lower detection than CFI and RMSEA. Under original categories, the 2-of-3 rule produced lower detection than the CFI-RMSEA AND rule. With collapsed categories, detection for the 2-of-3 rule increased.

The CFI-RMSEA OR acceptance rule provided a contrast in which only one component index was required to meet its calibrated threshold. Relative to the AND rule, the OR rule produced lower false rejection but also lower detection, especially in small-sample, moderate-loading scenarios. For example, under WLSMV with original categories, *N* = 250, and moderate loadings, the OR rule achieved lower false rejection than the AND rule but also lower detection for both Local and Severe Misfit. The OR rule therefore produced lower false rejection at the cost of lower detection, whereas the AND rule produced higher detection.

### Effects of collapsing on cutoffs and operating characteristics

**Fig. 5:**
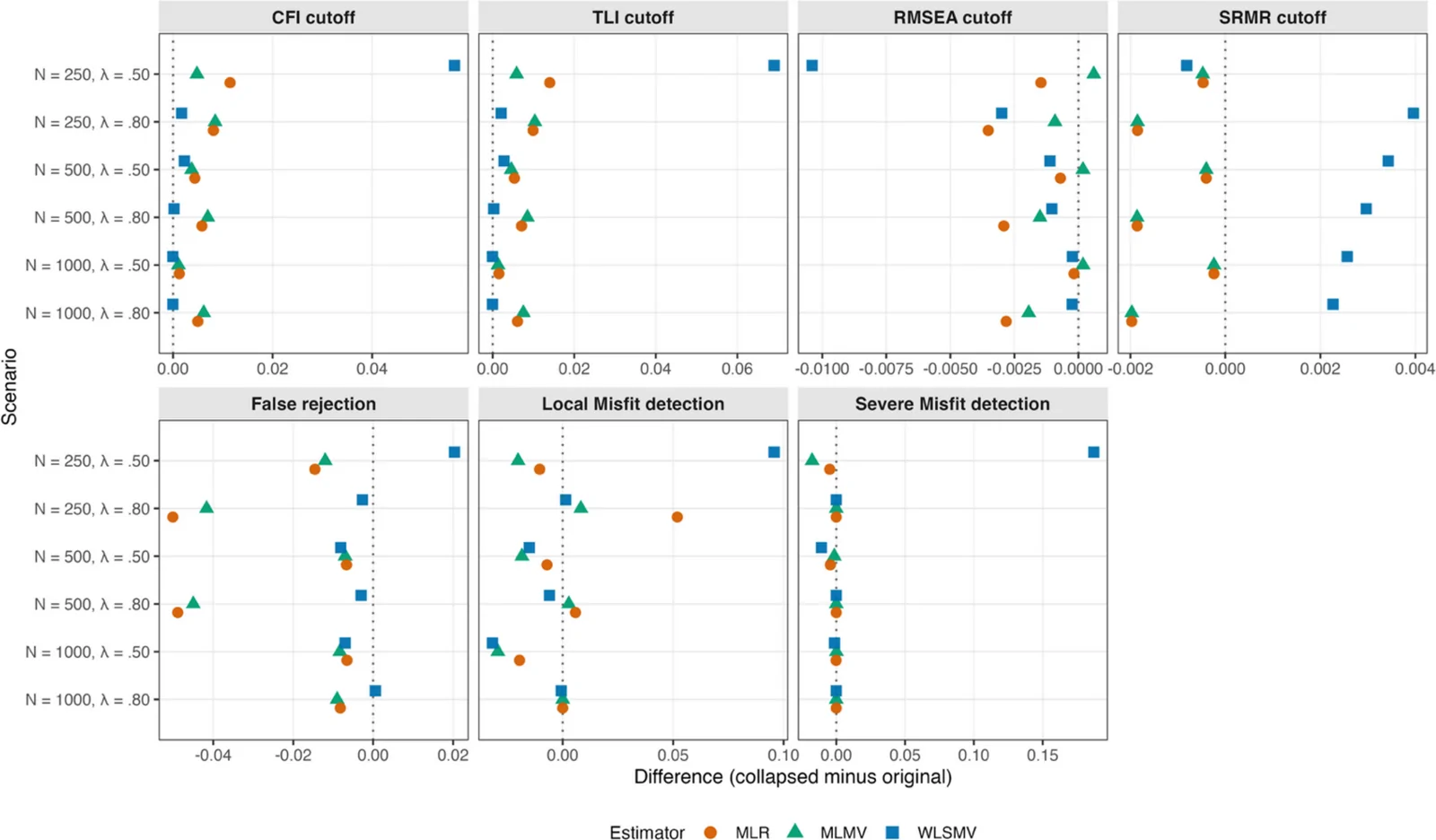
Effects of category collapsing on calibrated cutoffs and operating characteristics. *Note.* Paired collapsed-minus-original differences in calibrated cutoffs and operating characteristics. Differences were computed within each estimator, sample size, loading regime, sparsity pattern, and sparse-item-scope condition. Positive values indicate larger values after collapsing sparse response categories relative to retaining the original five-category response scale; negative values indicate smaller values after collapsing. The upper row shows differences in scenario-calibrated cutoffs for CFI, TLI, RMSEA, and SRMR. The lower row shows differences in CFI-RMSEA AND rule operating characteristics, including false rejection under the Correct model, detection under Local Misfit, and detection under Severe Misfit. The vertical dotted line marks no difference between collapsed and original analyses. The *x*-axis scale varies across panels. CFI = comparative fit index; TLI = Tucker–Lewis index; RMSEA = root mean square error of approximation; SRMR = standardized root mean square residual

Because original and collapsed analyses were paired within each sparse-data scenario, Fig. 5 summarizes the net change associated with moving from the sparse original five-category response scale to the corresponding collapsed response scale. Across MLR and MLMV, these paired contrasts were generally small, especially at larger sample sizes and under high loadings. For WLSMV, the paired contrasts were more pronounced in the small-sample, moderate-loading scenarios. In these settings, collapsing increased detection for several decisions while false-rejection rates remained relatively similar. These patterns show that the impact of collapsing depends jointly on estimator, sample size, and loading strength.

The calibrated-cutoff contrasts followed the same general pattern. For robust ML estimators, collapsed-minus-original cutoff differences were typically small, indicating that retaining or collapsing sparse categories produced similar cutoff locations in many scenarios. For WLSMV, cutoff shifts were more visible in the small-*N*, moderate-loading settings, where collapsing changed both incremental-index and absolute-index thresholds. Together, the cutoff and operating-characteristic contrasts show that the largest category-handling differences occurred for WLSMV in lower-information settings, whereas differences were generally smaller for robust ML estimators and in larger-sample or high-loading scenarios.

### Trimming extreme sparsity scenarios

To assess the influence of the most extreme sparse-category configurations, we trimmed the most extreme sparsity tier and recomputed cutoffs and operating characteristics. The cutoff and operating-characteristic sensitivity results are summarized in Table S3. Although some individual operating-characteristic changes were larger under the most severe sparsity conditions, the main estimator, sample-size, loading, and category-handling patterns remained stable after trimming.

### Prediction equations for scenario-specific cutoffs

Quantile regression coefficients for predicting scenario-level cutoffs are reported in Table 4. The prediction equations were intended as an approximate interpolation aid when an exact scenario match was unavailable. Consistent with the descriptive cutoff patterns, log_10_(*N*) was positively associated with CFI and TLI cutoffs and negatively associated with RMSEA and SRMR cutoffs, indicating that larger samples were associated with higher incremental-index cutoffs and smaller absolute-misfit cutoffs. Estimator contrasts were small for CFI, TLI, and RMSEA, whereas MLR and MLMV yielded lower SRMR cutoffs than WLSMV. Category handling showed small average associations with predicted cutoffs after accounting for estimator, loading regime, and sample size.

**Table 4:** Quantile regression coefficients for scenario-calibrated Correct-model cutoffs

Index	Predictor	*b*	*SE*	*p*
CFI (τ = .05)	Intercept	0.525	0.082	<.001
CFI	Collapse (1 = collapsed)	0.006	0.010	.533
CFI	MLR (vs. WLSMV)	0.004	0.016	.778
CFI	MLMV (vs. WLSMV)	0.004	0.015	.778
CFI	Loading (1 = .80, 0 = .50)	0.040	0.088	.654
CFI	log_10_(*N*)	0.141	0.045	.004
TLI (τ = .05)	Intercept	0.420	0.100	<.001
TLI	Collapse (1 = collapsed)	0.007	0.013	.554
TLI	MLR (vs. WLSMV)	0.005	0.021	.792
TLI	MLMV (vs. WLSMV)	0.005	0.019	.786
TLI	Loading (1 = .80, 0 = .50)	0.049	0.107	.650
TLI	log_10_(*N*)	0.172	0.055	.004
RMSEA (τ = .95)	Intercept	0.115	0.013	<.001
RMSEA	Collapse (1 = collapsed)	− 0.003	0.002	.192
RMSEA	MLR (vs. WLSMV)	− 0.003	0.004	.456
RMSEA	MLMV (vs. WLSMV)	− 0.004	0.004	.294
RMSEA	Loading (1 = .80, 0 = .50)	0.010	0.007	.176
RMSEA	log_10_(*N*)	− 0.032	0.004	<.001
SRMR (τ = .95)	Intercept	0.171	0.013	<.001
SRMR	Collapse (1 = collapsed)	− 0.001	0.002	.721
SRMR	MLR (vs. WLSMV)	− 0.013	0.004	.005
SRMR	MLMV (vs. WLSMV)	− 0.013	0.004	.005
SRMR	Loading (1 = .80, 0 = .50)	− 0.004	0.010	.695
SRMR	log_10_(*N*)	− 0.043	0.005	<.001

Models were fitted to scenario-level cutoffs calibrated at = α .05 from the Correct-model reference distributions. Scenario-level cutoffs were averaged across sparsity cells within each Estimator × Category handling × Sample size × Loading scenario. Cutoffs for CFI and TLI were modeled at the lower-tail 5th percentile τ = .05; cutoffs for RMSEA and SRMR were modeled at the upper-tail 95th percentile τ = .95. WLSMV, original category handling, and moderate loadings = λ .50 served as the reference categories. Collapse was coded 1 for the collapsed response scale and 0 for the original five-category response scale. Loading was coded 1 for high loadings = λ .80 and 0 for moderate loadings λ = .50; log_10_(*N*) is the base − 10 logarithm of sample size. For CFI and TLI, positive coefficients indicate higher cutoffs. For RMSEA and SRMR, positive coefficients also indicate higher cutoffs. CFI = comparative fit index; TLI = Tucker–Lewis index; RMSEA = root mean square error of approximation; SRMR = standardized root mean square residual; MLR = robust maximum likelihood; MLMV = mean and variance adjusted maximum likelihood; WLSMV = weighted least squares mean and variance adjusted

Prediction accuracy is summarized in Table S4 using absolute errors computed both in-sample and under leave-one-scenario-out cross-validation, where each held-out scenario was defined at the Estimator × Category handling × *N* × Loading level. Under the log_10_(*N*) specification, the leave-one-scenario-out median absolute error was approximately .053 for CFI, .065 for TLI, and .005 for RMSEA and SRMR. These cross-validation results indicate that the prediction equations are best suited to rough interpolation when an exact scenario match is unavailable. The scenario-specific cutoff tables provide a more direct reference when a close match exists. For applications with substantially different model structures or response formats, a tailored scenario-specific simulation provides a closer reference.

### Estimator-specific category-handling patterns

Table 5 summarizes estimator-specific cutoffs and operating characteristics for the CFI-RMSEA AND rule. Under moderate loadings, the rule showed a mean false-rejection rate of approximately .08 across estimator and category-handling conditions. Local Misfit detection was consistently lower than Severe Misfit detection, indicating that the omitted-cross-loading condition provided a more targeted sensitivity assessment than the two-factor Severe Misfit condition. In the moderate-loading panel, Local Misfit detection averaged .391, whereas Severe Misfit detection averaged .903.

**Table 5:** Estimator-specific summary of scenario-calibrated cutoffs and operating characteristics for the CFI-RMSEA AND rule

Panel	Estimator	Category handling	Mean CFI cutoff	Mean RMSEA cutoff	Mean SRMR cutoff	AND false rejection	AND Local Misfit detection	AND Severe Misfit detection	No. scenarios	Category-handling pattern
Panel A. Moderate loadings only	MLR	Original	.939	.027	.038	.088	.382	.904	3	Original sparse categories retained
	MLR	Collapsed	.944	.026	.038	.079	.370	.900	3	Collapsing modestly reduced false rejection, with similar Local and Severe Misfit detection
	MLMV	Original	.944	.023	.038	.087	.397	.913	3	Original sparse categories retained
	MLMV	Collapsed	.947	.024	.038	.078	.375	.907	3	Collapsing mainly reduced false rejection; detection was similar
	WLSMV	Original	.946	.028	.045	.072	.403	.867	3	Original sparse categories retained
	WLSMV	Collapsed	.966	.024	.047	.074	.419	.925	3	Collapsing increased detection for WLSMV, especially under moderate loadings
Panel B. Overall average across loadings	MLR	Original	.958	.030	.037	.144	.644	.952	6	Original sparse categories retained
	MLR	Collapsed	.964	.028	.036	.122	.648	.950	6	Collapsing mainly reduced false rejection; detection was similar
	MLMV	Original	.960	.026	.037	.144	.662	.957	6	Original sparse categories retained
	MLMV	Collapsed	.965	.025	.036	.123	.653	.954	6	Collapsing mainly reduced false rejection; detection was similar
	WLSMV	Original	.970	.027	.040	.072	.667	.933	6	Original sparse categories retained
	WLSMV	Collapsed	.981	.024	.042	.072	.674	.963	6	Collapsing mainly increased detection in lower-information settings

Cutoffs are scenario-averaged values calibrated at α = .05 from the Correct-model reference distributions. AND false rejection is the rate under the Correct fitted model; Local Misfit detection is the rate under the omitted-cross-loading model; Severe Misfit detection is the rate under the two-factor model combining Factors 1 and 2. Panel A averages *N* = 250, 500, and 1,000 under moderate loadings; Panel B averages the same sample sizes across both loading regimes. CFI = comparative fit index; RMSEA = root mean square error of approximation; SRMR = standardized root mean square residual; WLSMV = weighted least squares mean and variance adjusted

The category-handling patterns differed by estimator family. For MLR and MLMV, collapsing sparse categories modestly reduced false rejection while producing similar Local and Severe Misfit detection. In the overall averages across both loading regimes, false rejection for the CFI-RMSEA AND rule decreased from .144 to .122 for MLR and from .144 to .123 for MLMV, while detection remained similar across the two response-scale versions. For WLSMV, the clearest change was in detection. In the moderate-loading panel, Severe Misfit detection increased from .867 under original categories to .925 under collapsed categories, with little change in false rejection. Across both loading regimes, Severe Misfit detection increased from .933 to .963. These patterns show different category-handling trade-offs across estimators, with the largest detection increases for WLSMV in lower-information conditions.

### Sensitivity and robustness checks

Sensitivity analyses produced broadly similar directional patterns across the alternative specifications (Table S3). Recalibration at α = .01 and α = .10 produced the corresponding shifts in the cutoffs, with the same directional dependencies on estimator, sample size, loading strength, and category handling. Trimming the most extreme sparsity tier produced modest average changes in scenario-level cutoffs and operating characteristics, although some individual operating-characteristic changes were larger under the most severe sparsity conditions. The realized false-rejection profiles in Table S3, Panel C, further show that composite rules and single indices differed in their empirical deviation from the nominal.05 reference level. Across these checks, fit-index reference distributions remained scenario-dependent, Local Misfit detection remained lower than Severe Misfit detection, the CFI-RMSEA AND rule retained a similar operating profile, and category collapsing continued to have its largest effects for WLSMV in lower-information settings.

The next section applies the same calibration logic to an empirical sparse ordinal scale with a different measurement structure.

## Applied illustration

To illustrate the calibration workflow in an empirical setting, we analyzed six household welfare perception items from the Sierra Leone baseline household survey (L. Liu, Okech, & Cody, 2026; Okech et al., 2022). Unlike the 15-item three-factor model used in the main simulation, the applied example uses a six-item one-factor ordinal CFA with its own empirical response distributions. We therefore document the sparse response structure, compare original and collapsed category handling, evaluate conventional CFA fit, and construct a model-conditional reference distribution through a tailored mini-simulation.

### Dataset and item set

The applied example used six five-category ordinal items from the Sierra Leone baseline household survey. The survey was conducted in Kailahun, Kenema, and Kono and included 3,070 sampled households (Okech et al., 2022). The applied illustration focused on the household welfare perception items in the survey, which asked respondents whether the household was well off, found it difficult to live on current income, had enough food, had enough money for school fees, had enough money for clothing, and had enough money for medicine. Items were oriented so that higher values indicated greater household economic strain. The income-difficulty item was reverse-coded before analysis so that the direction of the scale was consistent across items. Analyses used complete cases on the six items, yielding an analytic sample of *N* = 2,989.

The CFA model specified one latent household economic strain factor measured by the six ordinal indicators. This structure differs from the main simulation in its number of factors, number of items, and empirical response distributions. Given these differences, we constructed a model-conditional reference distribution directly for the six-item one-factor model using the same calibration logic.

### Sparsity diagnostics and category handling

We first summarized marginal and bivariate sparsity for the original five-category response scale. As shown in Table 6, the original response scale exhibited clear sparse ordinal structure. Five of the six items had at least one sparse marginal category. Across the 15 bivariate item tables, there were 13 zero cells and 110 cells with fewer than five observations. These diagnostics indicate that the original five-category scale contained sparse marginal and bivariate response patterns that could affect ordinal CFA estimation and fit-index behavior.

**Table 6:** Applied illustration summary for the Sierra Leone household welfare items

Panel A. Observed sparsity diagnostics and WLSMV CFA fit																			
Response scale	*N*	Sparse marginal categories				Bivariate zero cells		Bivariate cells < 5		${\upchi }^{2}(df)$	*p*		CFI	TLI	RMSEA [90% CI]		SRMR		Standardized loading range
Original 5-category	2,989	5 of 6 items				13		110		177.204 (9)	<.001		.998	.996	.079 [.069, .089]		.012		.351 to .933
Collapsed 3-category	2,989	0 of 6 items				0		0		96.239 (9)	<.001		.998	.997	.057 [.047, .067]		.019		.248 to .952
Panel B. Conventional and scenario-calibrated interpretations																			
Response scale				Index	Observed				Conventional benchmark (result)						Scenario-calibrated cutoff				
Original 5-category				CFI	.998				≥ .950 (meets)						≥ .999818				
Original 5-category				TLI	.996				≥ .950 (meets)						≥ .999697				
Original 5-category				RMSEA	.079				≤ .060 (Does not meet)						≤ .016881				
Original 5-category				SRMR	.012				≤ .080 (meets)						≤ .006220				
Collapsed 3-category				CFI	.998				≥ .950 (meets)						≥ .999805				
Collapsed 3-category				TLI	.997				≥ .950 (meets)						≥ .999676				
Collapsed 3-category				RMSEA	.057				≤ .060 (meets)						≤ .016434				
Collapsed 3-category				SRMR	.019				≤ .080 (meets)						≤ .009441				
Panel C. Sample-size sensitivity of model-conditional calibrated cutoffs																			
Response scale			*N*				CFI cutoff			TLI cutoff		RMSEA cutoff				SRMR cutoff		Available replications	
Original 5-category			500				≥ .998896			≥ .998159		≤ .042765				≤ .015553		953	
Original 5-category			1,000				≥ .999432			≥ .999054		≤ .029851				≤ .010642		997	
Original 5-category			2,989				≥ .999818			≥ .999697		≤ .016881				≤ .006220		1,000	
Collapsed 3-category			500				≥ .998820			≥ .998033		≤ .041349				≤ .023496		1,000	
Collapsed 3-category			1,000				≥ .999398			≥ .998997		≤ .029848				≤ .017197		1,000	
Collapsed 3-category			2,989				≥ .999805			≥ .999676		≤ .016434				≤ .009441		1,000	

The WLSMV CFA specified a single household economic strain factor. Tailored mini-simulations used the fitted one-factor model as a model-conditional plug-in reference. Panel B reports calibrated cutoffs for the empirical sample size *N* = 2,989; Panel C reports sensitivity results for *N* = 500, *N* = 1,000, and *N* = 2,989, with fitted loadings, thresholds, response-scale specification, and estimator held fixed. In Panel B, all observed fit-index values fell on the worse-fit side of their respective model-conditional calibrated cutoffs. CFI and TLI cutoffs correspond to the lower 5th percentile whereas RMSEA and SRMR cutoffs correspond to the upper 95th percentile of the model-conditional reference distributions. A total of 1,000 replications were requested per condition; the original five-category conditions yielded 953 available replications at *N* = 500 and 997 available replications at *N* = 1,000 because some replications contained an empty sparse response category. All other conditions yielded 1,000 available replications. The calibrated cutoffs are conditional on the working reference model and apply to the specified reference scenario. = ${\upchi }^{2}$chi-square test statistic; CFI = comparative fit index; TLI = Tucker–Lewis index; RMSEA = root mean square error of approximation; SRMR = standardized root mean square residual; WLSMV = weighted least squares mean and variance adjusted

We then created a collapsed three-category version of the scale. After aligning item direction, the two lower response categories were combined, the middle category was retained, and the two upper response categories were combined. This deterministic recoding reduced sparse response structure in the empirical data. In the collapsed three-category version, no items had sparse marginal categories, and the bivariate item tables contained no zero cells and no cells with fewer than five observations. Thus, the original and collapsed versions provided a paired applied contrast between retaining the sparse five-category response scale and using a collapsed response scale with improved marginal and bivariate cell counts.

### Conventional CFA fit

For both response-scale versions, we fitted a one-factor WLSMV CFA model and recorded the chi-square test of model fit together with CFI, TLI, RMSEA, and SRMR. For the original five-category scale, the chi-square test rejected exact fit, ${\upchi }^{2}(9)\hspace{0.17em}=$ 177.204, *p* < .001. The descriptive fit indices were CFI = .998, TLI = .996, RMSEA = .079, 90% CI [.069, .089], and SRMR = .012. Thus, CFI, TLI, and SRMR met the conventional heuristic benchmarks, whereas RMSEA exceeded the .06 benchmark. For the collapsed three-category scale, the chi-square test also rejected exact fit, ${\upchi }^{2}(9)$ = 96.239, *p* < .001. The descriptive fit indices were CFI = .998, TLI = .997, RMSEA = .057, 90% CI [.047, .067], and SRMR = .019. Under the conventional benchmarks considered here, all four descriptive indices met their corresponding heuristic criteria after collapsing, despite rejection of exact fit by the chi-square test. This contrast illustrates the complementary roles of the chi-square test and descriptive fit indices: the former evaluates exact fit, whereas the latter summarize different aspects of approximate, relative, or residual fit.

The standardized loadings varied substantially across items. Standardized loadings ranged from .351 to .933 in the original five-category version and from .248 to .952 in the collapsed three-category version. The income-difficulty item had the weakest loading in both response-scale versions. Collapsing removed the sparse-cell diagnostics and reduced RMSEA, while the spread in standardized loadings indicated substantial item-level heterogeneity.

### Scenario-specific mini-simulation calibration and interpretation

To illustrate scenario-specific calibration for the empirical setting, we treated the fitted one-factor WLSMV model as a model-conditional plug-in reference for both response-scale versions. Its estimated standardized loadings and thresholds defined the working data-generating structure. The resulting reference distributions characterize the expected sampling behavior of CFI, TLI, RMSEA, and SRMR under this model and parameterization. Their interpretation is therefore conditional on the fitted model serving as an adequate working approximation to the population structure.

For each response-scale version, we generated 1,000 replications and fitted the corresponding one-factor WLSMV CFA model. The primary calibration retained the empirical sample size of *N* = 2,989. Cutoffs were obtained from the resulting model-conditional reference distributions using the same tail-quantile logic as in the main simulation. At α = .05, CFI and TLI cutoffs were defined by the lower-tail 5th percentile, whereas RMSEA and SRMR cutoffs were defined by the upper-tail 95th percentile. Sample-size sensitivity at *N* = 500 and *N* = 1,000 is reported below.

At the empirical sample size of *N* = 2,989, the model-conditional reference distributions were highly concentrated near their boundary values. For the original five-category version, the calibrated cutoffs were CFI ≥ .999818, TLI ≥ .999697, RMSEA ≤ .016881, and SRMR ≤ .006220. For the collapsed three-category version, the corresponding cutoffs were CFI ≥ .999805, TLI ≥ .999676, RMSEA ≤ .016434, and SRMR ≤ .009441.

These calibrated values are conditional quantiles of the sampling distributions generated under a particular fitted reference model, estimator, response structure, and sample size. With *N* = 2,989, sampling variability around the plug-in reference model is relatively small, so the correct-model distributions are concentrated near their boundary values. The proximity of the calibrated cutoffs to 1.00 for CFI and TLI and to zero for RMSEA and SRMR therefore reflects the large-sample reference distribution for this specific scenario.

### Sample-size sensitivity

To illustrate the sample-size dependence of the model-conditional reference distributions, we repeated the plug-in calibration at *N* = 500 and *N* = 1,000, while holding the fitted loadings, thresholds, response-scale specification, WLSMV estimator, and number of requested replications fixed. The resulting calibrated cutoffs and available replication counts for *N* = 500, *N* = 1,000, and *N* = 2,989 are reported in Table 6, Panel C. For the original five-category scale, the calibrated CFI cutoff increased from .998896 at *N* = 500 to .999432 at *N* = 1,000 and .999818 at *N* = 2,989. The corresponding TLI cutoffs were .998159, .999054, and .999697. In the opposite direction, the RMSEA upper-tail cutoff decreased from .042765 to  .029851 and .016881, and the SRMR cutoff decreased from .015553 to .010642 and .006220. A similar pattern was observed for the collapsed three-category scale. The CFI cutoffs were .998820, .999398, and .999805 across *N* = 500, *N* = 1,000, and *N* = 2,989, respectively; the corresponding TLI cutoffs were .998033, .998997, and .999676. RMSEA cutoffs decreased from .041349 to .029848 and .016434, while SRMR cutoffs decreased from .023496 to .017197 and .009441.

Across both response-scale specifications, larger samples produced increasingly concentrated Correct-model reference distributions, with CFI and TLI cutoffs moving toward 1.00 and RMSEA and SRMR cutoffs moving toward zero. This pattern links the near-boundary cutoffs at *N* = 2,989 to the greater concentration of the large-sample reference distributions.

Taken together, the illustration shows how category handling, working-model specification, and sample size jointly shape scenario-specific calibration. Collapsing removed the sparse-cell diagnostics and reduced RMSEA from .079 to .057, placing the collapsed model below the conventional .06 benchmark, while the sample-size sensitivity analysis showed increasing concentration of the model-conditional reference distributions as *N* increased. The calibrated values are therefore most informative when interpreted together with the stated working model, conventional fit information, and diagnostic evidence.

## Discussion

This study extended scenario-specific simulation-based fit-index calibration to sparse ordinal CFA with category collapsing. We calibrated CFI, TLI, RMSEA, and SRMR cutoffs from Correct-model reference distributions and evaluated their operating characteristics under Correct, Local Misfit, and Severe Misfit fitted-model conditions. This design allowed us to examine three linked questions: how sparse ordinal response structures shift fit-index reference distributions, how single-index and composite decision rules behave under localized and stronger misspecification, and when category collapsing changes model-evaluation decisions.

Three broad findings emerged. First, scenario-calibrated cutoffs differed substantially from conventional fixed benchmarks. RMSEA and SRMR cutoffs were typically much lower than .06 and .08, respectively, whereas CFI and TLI cutoffs varied around .95 depending on sample size and loading strength. Second, detection depended strongly on the form of misspecification. Detection was consistently lower for Local Misfit than for Severe Misfit, indicating that evaluations based only on strong structural misspecification may provide an incomplete picture of sensitivity to more localized measurement misspecification. Third, category collapsing had estimator-specific effects. For robust ML estimators, collapsing mainly produced modest shifts in false rejection and detection. For WLSMV, the largest category-handling differences occurred in lower-information settings, especially when sparse bivariate information was limited.

### Scenario-specific cutoffs and estimator differences

The cutoff results show that sparse ordinal CFA changes the reference distributions of commonly reported fit indices. Conventional benchmarks such as CFI or TLI ≥ .95, RMSEA ≤ .06, and SRMR ≤ .08 did not align consistently with the scenario-calibrated Correct-model reference distributions. The most consistent divergence occurred for RMSEA and SRMR. Their scenario-calibrated cutoffs were often far below conventional values, so an index value that satisfies a conventional heuristic benchmark can still fall on the worse-fit side of the Correct-model reference distribution in these sparse ordinal scenarios.

Incremental-index cutoffs showed a different pattern. Under moderate loadings and small samples, calibrated CFI and TLI cutoffs could fall well below .95 because the correct-model distributions were more dispersed. Under high loadings or larger samples, the correct-model distributions were concentrated closer to the upper boundary, producing higher CFI and TLI cutoffs. This pattern highlights an important feature of bounded incremental indices: as sampling variability decreases and correct-model indices cluster near 1.00, small departures from the upper boundary become more consequential for percentile-based cutoffs. Thus, the same observed CFI or TLI value can carry different implications depending on sample size, loading strength, response sparsity, estimator, and category handling.

Estimator differences were also visible. MLR and MLMV produced broadly similar cutoff patterns, whereas WLSMV showed more distinct behavior, especially for incremental indices and in sparse small-sample conditions. These differences are consistent with the way WLSMV uses thresholds, polychoric correlations, and weight matrices, whereas robust ML estimators treat ordinal indicators as approximately continuous and rely on the observed mean and covariance structure of the discretized variables. These differences underscore the importance of accounting for estimator choice when calibrating fit-index reference values.

### Why sparsity changes fit-index behavior

The scenario-specific cutoff patterns can be understood through the information structure of sparse ordinal CFA. Sparse response patterns concentrate observations in a limited number of categories and leave some marginal or bivariate cells with little empirical information. For WLSMV, this directly affects the limited-information quantities used for estimation and model evaluation, including thresholds, polychoric correlations, and the weight matrix. Sampling variation in thresholds, polychoric correlations, and the weight matrix propagates into the model discrepancy function, baseline-model comparison, and residual association structure from which CFI, TLI, RMSEA, and SRMR are computed. This mechanism helps explain why WLSMV showed distinctive cutoff behavior and why collapsing sparse categories was most consequential in small-sample, moderate-loading scenarios.

For MLR and MLMV, sparsity operates through a different but related pathway. When ordinal indicators have highly asymmetric response distributions, the observed covariance structure of the discretized indicators can differ from the structure implied by balanced continuous indicators. Robust corrections adjust test statistics and standard errors, but fit indices remain functions of the fitted covariance structure, the baseline model, and discrepancies between observed and model-implied moments. Sparse and highly asymmetric response distributions can therefore shift correct-model fit-index distributions even when the factor structure is correctly specified and estimation converges. This pathway helps explain why robust ML estimators showed elevated false rejection in some high-loading scenario-level summaries, where fit indices became highly sensitive to small sample-to-sample discrepancies.

Category collapsing modifies these information pathways. It can stabilize estimation by moving observations out of extremely sparse categories and increasing effective marginal and bivariate cell counts. At the same time, it reduces the number of response distinctions available to the model and can change the information available for detecting localized misfit. The paired original-versus-collapsed contrasts in this study show how these forces combine in practice. Collapsing had modest effects for MLR and MLMV across many scenarios, whereas it produced clearer changes for WLSMV in lower-information settings. This pattern is consistent with an estimator-specific mechanism in which sparse contingency information plays a larger role for limited-information ordinal estimation than for robust continuous approximations.

### Local and severe misfit as complementary detection targets

The distinction between Local Misfit and Severe Misfit was central to evaluating decision-rule sensitivity. The Local Misfit condition omitted the prespecified cross-loading while retaining the three-factor structure. The Severe Misfit condition combined the first two factors into a two-factor structure. These two fitted-model conditions provide complementary detection targets. Local Misfit represents a targeted measurement-structure departure that may resemble common applied misspecifications, such as omitting a secondary loading. Severe Misfit represents a stronger structural departure and provides a contrasting detection target.

The observed detection rates reflected this contrast. Across decision rules, Local Misfit detection was consistently lower than Severe Misfit detection. For example, under moderate loadings, the CFI-RMSEA AND rule yielded an average Local Misfit detection rate of .391 and an average Severe Misfit detection rate of .903. Across both loading regimes, the corresponding averages were .658 and .951. These patterns show that fit-index operating characteristics depend strongly on the type and strength of misspecification used for evaluation. Together, the two conditions show how operating characteristics change across localized and stronger forms of misspecification. Severe Misfit provides a benchmark for detection under a stronger structural departure, whereas Local Misfit provides a more targeted assessment of sensitivity to measurement-level departures.

### Decision rules and empirical error profiles

The composite-rule results show how scenario-calibrated indices behave when interpreted jointly. The CFI-RMSEA AND rule pairs an incremental-fit index with an absolute-misfit index using their calibrated component thresholds. In moderate-loading scenarios, this rule generally yielded higher detection than the component indices, while false rejection remained within a relatively narrow range.

Under moderate loadings, its average false-rejection rate was .080 across estimator and category-handling scenarios. Local Misfit detection averaged .391, and Severe Misfit detection averaged .903. Across the full grid, including both moderate and high loadings, the average false-rejection rate increased to .113, with Local Misfit detection averaging .658 and Severe Misfit detection averaging .951. This broader variation was concentrated primarily in high-loading robust ML scenarios, where CFI and RMSEA were especially sensitive to small discrepancies. These results show that composite rules can be evaluated directly as decision tools with observable trade-offs between false rejection under correct specification and detection under localized or severe misspecification.

The 2-of-3 rule, based on CFI, RMSEA, and SRMR, provided a majority-style comparison. Its performance was often similar to the CFI-RMSEA AND rule for MLR and MLMV, especially when sample size was at least 500. In lower-information WLSMV settings, SRMR’s higher false rejection and lower detection attenuated the performance of the 2-of-3 rule relative to the CFI-RMSEA AND rule. The CFI-RMSEA OR acceptance rule provided a contrast in which only one component index was required to meet its cutoff. It reduced false rejection but also reduced detection, especially in small-sample moderate-loading conditions. SRMR added residual-based information but showed a less stable decision profile in lower-information WLSMV scenarios.

The composite-rule results also point to a future extension. The present rules combined marginally calibrated component cutoffs, providing an interpretable framework for joint fit-index decisions. Future work can extend this approach by jointly calibrating multivariate decision boundaries, such as CFI-RMSEA regions selected to target a prespecified overall operating characteristic. Such work would move from marginal component calibration to direct calibration of the composite rule itself.

### Category collapsing and estimator-specific guidance

The effects of category collapsing varied by estimator and scenario. Original and collapsed analyses were paired within each sparse-data scenario, so the contrasts summarize the net change associated with moving from sparse retained categories to the corresponding collapsed response scale. This comparison reflects an applied decision researchers often face when sparse ordinal categories are present.

For MLR and MLMV, collapsing generally produced small changes in calibrated cutoffs and operating characteristics. The most consistent change was a modest reduction in false rejection, with Local and Severe Misfit detection remaining similar across original and collapsed response scales. In the overall averages for the CFI-RMSEA AND rule, false rejection decreased from .144 to .122 for MLR and from .144 to .123 for MLMV when categories were collapsed. Little change in detection was found for either robust ML estimator.

For WLSMV, collapsing showed a different profile. False rejection for the CFI-RMSEA AND rule was essentially unchanged in the overall averages, whereas detection increased. Severe Misfit detection increased from .933 under original categories to .963 under collapsed categories across both loading regimes. Under moderate loadings, Severe Misfit detection increased from .867 to .925. The largest detection increases occurred in lower-information settings where sparse bivariate information was most consequential. When response categories carry substantive meaning and estimation is stable, retaining the original scale preserves the original response distinctions. When extreme categories are empty or nearly empty, collapsing can increase effective cell counts and may stabilize ordinal estimation, particularly for WLSMV in small samples.

### Applied illustration

The applied illustration demonstrated how the scenario-specific calibration workflow can be implemented for a real sparse ordinal scale. The six Sierra Leone household welfare items exhibited the sparse response features that motivated the simulation study, including sparse marginal categories and sparse bivariate tables under the original five-category response scale. Collapsing to three categories removed these sparse-cell diagnostics and reduced RMSEA from .079 to .057, bringing the collapsed model below the conventional .06 benchmark.

The example also showed that conventional benchmarks and model-conditional reference distributions can lead to different interpretations. Under the collapsed three-category scale, all four conventional fit-index benchmarks were met, whereas the observed indices fell on the worse-fit side of the calibrated thresholds from the working-model reference distribution. This contrast illustrates how an explicitly specified reference scenario can provide a conditional benchmark alongside conventional fit information. Its interpretation therefore depends on how well the working reference model represents the empirical structure.

The main simulation tables are most informative for scenarios resembling the simulated design. For settings with different item sets, response structures, sample sizes, or estimators, the same calibration logic can be used to construct a model-conditional reference distribution based on a substantively plausible working model.

### Practical workflow for applied researchers

The results suggest a practical workflow for sparse ordinal CFA. Researchers should first document the response structure before interpreting model fit, including marginal category proportions, bivariate sparse cells, the number of items affected by sparse categories, and whether sparsity is concentrated in extreme response options. They should then specify the estimator and category-handling strategy because both factors affect the Correct-model reference distributions and the operating characteristics of cutoff-based decisions. When the empirical design closely resembles the simulated conditions, the closest scenario-specific cutoff table can provide a scenario-matched descriptive reference. When the model structure, number of items, response scale, sample size, or sparsity mechanism differs substantially from the simulation grid, a tailored scenario-specific simulation provides a closer model-conditional reference.

An empirical reference model should be both substantively plausible and empirically adequate. Before calibration, researchers should evaluate the working model using theory, local fit diagnostics, residual patterns, and plausible alternative specifications. If several specifications remain plausible, the calibration can be repeated across them to examine the sensitivity of the resulting reference values. The resulting cutoffs are then interpreted as conditional on the stated working model and parameterization. The prediction equations are best viewed as rough interpolation aids. When a close simulation match is available, the corresponding scenario-level cutoff table provides the more direct reference. For substantially different empirical designs, a tailored simulation provides a closer model-conditional reference.

Fit decisions should be reported together with the relevant decision rule and operating-characteristic profile. Scenario-calibrated descriptive fit indices are one component of a broader model-fit evaluation. Consistent with recommendations for comprehensive SEM fit evaluation, researchers should report the model chi-square test together with descriptive fit indices and interpret the two according to their distinct roles: the former as a test of exact fit and the latter as summaries of approximate, relative, or residual fit (Zheng & Bentler, 2025). A clear report would pair the selected decision rule with its scenario-level false-rejection and detection rates. Category collapsing should also be reported as a modeling decision with both statistical and substantive implications. Collapsing can increase effective cell counts and stabilize ordinal estimation, but it also changes the response scale. In the simulated conditions, the largest category-handling differences occurred when sparsity was severe and WLSMV was used, especially in lower-information settings. When categories carry substantive meaning and estimation is stable, retaining the original response scale preserves the original response distinctions.

### Relation to prior work

The present findings reinforce and extend several strands of the model-fit literature. First, they align with long-standing critiques of universal fit-index cutoffs. Prior work has shown that fit indices depend on sample size, loading strength, model complexity, estimator choice, and distributional features of the indicators (Chen et al., 2008; Fan & Sivo, 2007; Kenny & McCoach, 2003; Marsh et al., 2004; Moshagen, 2012; Shi et al., 2019). The present study extends this evidence to sparse ordinal CFA with category collapsing and shows that sparse response patterns can substantially alter correct-model reference distributions for commonly reported indices.

Second, this study extends scenario-specific simulation-based cutoff approaches by applying them to a sparse ordinal setting and by evaluating both single-index decisions and transparent composite rules. This connects the simulation-based calibration logic described by Groskurth et al. (2024), McNeish and Wolf (2023), Millsap (2007, 2013), Niemand and Mai (2018), and Pornprasertmanit (2014) to a common applied problem: interpreting CFA fit when ordinal response categories are sparse and category collapsing is under consideration. The results show that scenario-specific calibration yields both cutoff values and corresponding operating-characteristic summaries. These findings also complement recent simulation-cum-ROC evidence showing that diagnostic discrimination under sparse ordinal category collapsing depends on the misspecification target and information conditions (L. Liu et al., 2026). Here, we address the complementary calibration problem by deriving cutoffs from Correct-model reference distributions and evaluating the operating characteristics of the resulting decision rules.

Third, the findings broaden the literature on sparse ordinal category handling. Prior work has often focused on estimation stability, convergence, and the performance of categorical versus continuous estimation strategies (DiStefano et al., 2021; Flora & Curran, 2004; Forero et al., 2009; Rhemtulla et al., 2012; Savalei, 2012; Xia & Yang, 2019). The present study adds that category handling also affects fit-index reference distributions and decision-rule behavior. Thus, collapsing sparse categories has implications not only for estimation but also for model-evaluation decisions.

Fourth, the SRMR results connect with recent work on population-targeted and bias-corrected fit assessment. Conventional sample-based SRMR showed less stable decision performance in several sparse ordinal settings, especially for WLSMV in lower-information conditions. This pattern is consistent with the broader argument that residual fit measures can reflect sample behavior, estimator-specific scaling, and distributional features in addition to substantive misfit. This connection motivates future comparisons between conventional SRMR and unbiased or population-oriented residual fit measures under sparse ordinal category structures.

### Strengths, boundary conditions, and future directions

This study has several strengths. It used a fully crossed simulation design that varied estimator, sample size, loading strength, sparsity pattern, sparse-item scope, and category handling. It calibrated cutoffs from Correct-model reference distributions and evaluated operating characteristics under both Local and Severe Misfit. It also examined single-index and composite decision rules, quantified Monte Carlo uncertainty for operating-characteristic estimates, and added an applied illustration showing how the workflow can be implemented for a real sparse ordinal scale.

Our analyses center on conventional software-reported fit indices, which remain common in applied CFA reporting and cutoff-based decisions. Population-targeted and bias-corrected measures address a related aspect of fit assessment. Direct comparisons between conventional SRMR and unbiased or population-oriented residual fit measures under the same sparse-category conditions remain an important direction for future research.

The results are most directly applicable to settings resembling the simulated design: a three-factor ordinal CFA with five-category indicators, the studied range of sample sizes and loading strengths, and sparse response patterns similar to those represented in the simulation grid. The use of a fixed population structure provided a common target for examining how sparsity, estimator, loading strength, sample size, and category handling shape fit-index reference distributions. The resulting cutoffs are therefore tied to the simulated model structure and analysis conditions. Different factor structures, numbers of items, factor correlations, response scales, sparsity mechanisms, estimators, or misspecification types may produce different reference distributions and operating characteristics. The tabulated cutoffs are therefore most informative for applications that closely resemble the simulated structure. For substantially different designs, the tables can provide a rough orientation, while a tailored scenario-specific simulation offers a closer reference. Monte Carlo uncertainty was quantified for the scenario-level operating characteristics based on the available replication counts. Larger replication counts would further reduce Monte Carlo uncertainty, particularly for operating rates near the boundaries of 0 or 1.

Several extensions follow directly from the present findings. First, future work can jointly calibrate multivariate decision rules by selecting CFI-RMSEA or multi-index decision regions that target a prespecified overall operating characteristic. This would extend the present marginal-threshold approach to composite rules calibrated as complete decision procedures. Second, additional misspecification conditions can be examined, including correlated residuals, multiple omitted cross-loadings, factor correlation misspecification, threshold non-invariance, and partial scalar misspecification. These conditions would clarify how sensitivity changes across localized, moderate, and structural forms of misfit. Third, simulation grids can be expanded to additional sample-size ranges, loading distributions, factor correlations, item counts, and response-scale formats. Fourth, future studies can compare conventional software-reported fit indices with population-targeted or bias-corrected alternatives, especially unbiased SRMR and related residual-based fit measures. Fifth, the scenario-specific calibration workflow can be extended to Bayesian SEM, multilevel SEM, multigroup ordinal CFA, and longitudinal ordinal measurement models.

## Conclusion

Sparse ordinal CFA produces fit-index reference distributions that can differ substantially from conventional fixed benchmarks. In the present simulations, RMSEA and SRMR calibrated cutoffs were generally much lower than conventional values, and CFI and TLI cutoffs varied with sample size and loading strength. Detection depended strongly on the form of misspecification, with consistently lower detection for Local Misfit than for Severe Misfit. The CFI-RMSEA AND rule yielded an interpretable empirical error profile across the simulated scenarios. SRMR showed a distinct residual-based profile, but its decision-rule operating characteristics were less stable in lower-information sparse ordinal settings, especially for WLSMV. Category collapsing had estimator-specific effects, with the largest detection gains for WLSMV in lower-information conditions. Together, the simulation and applied illustration support a scenario-specific approach to fit-index interpretation, in which researchers document sparse response structure, select cutoffs matched to the estimator and design, and report the operating-characteristic profile of the decision rule used.

## Supplementary Information

Below is the link to the electronic supplementary material.

Supplementary file1 (659 KB PDF)

## Data Availability

The de-identified analytic dataset containing the six household economic strain items used in the applied illustration, together with the simulation outputs and supplementary results, is publicly available in the Open Science Framework repository at https://osf.io/ce7rm/.

## Code availability

Simulation and analysis code for this study are publicly available in the Open Science Framework repository at https://osf.io/ce7rm/.
