# Supplementary material for: Calibrating fit-index cutoffs for sparse ordinal CFA: Scenario-specific thresholds and operating characteristics
Source: Behav Res Methods. 2026 Nov 4;58(12):341. doi: 10.3758/s13428-026-03221-8 (PMC13736407; doi:10.3758/s13428-026-03221-8)
Supplement: Supplementary file 1 [file 13428_2026_3221_MOESM1_ESM.pdf]

Calibrating Fit-Index Cutoffs for Sparse Ordinal CFA: Scenario-Specific Thresholds and Operating Characteristics

Supplementary Material

Table S1

Replication Diagnostics for Simulation Conditions With Incomplete or Flagged Replications

Estimator	Handling	N	Loading	Scope	Pattern	Fitted model	Available R	Missing FI R	Empty-cat. R	Nonconv. R	Improper R	Admissible R
MLR	Original	250	Moderate	4I	4C	Correct	989	0	0	11	37	952
MLR	Original	250	Moderate	4I	4C	Local Misfit	999	0	0	1	46	953
MLR	Original	250	Moderate	4I	4C	Severe Misfit	999	0	0	1	3	996
MLR	Original	250	High	1I	1C	Severe Misfit	1000	1	0	0	0	999
MLR	Original	250	High	2I	2C	Severe Misfit	1000	1	0	0	0	999
MLR	Original	250	High	4I	4C	Severe Misfit	1000	2	0	0	0	998
MLR	Original	500	Moderate	4I	4C	Correct	999	0	0	1	1	998
MLR	Collapsed	250	Moderate	4I	4C	Correct	989	0	0	9	24	965
MLR	Collapsed	250	Moderate	4I	4C	Local Misfit	998	0	0	2	32	966
MLR	Collapsed	250	Moderate	4I	4C	Severe Misfit	999	0	0	1	3	996
MLR	Collapsed	250	High	1I	1C	Severe Misfit	1000	1	0	0	0	999
MLR	Collapsed	500	High	2I	2C	Severe Misfit	1000	1	0	0	0	999
MLMV	Original	250	Moderate	4I	4C	Correct	989	0	0	11	37	952
MLMV	Original	250	Moderate	4I	4C	Local Misfit	999	0	0	0	47	953
MLMV	Original	250	Moderate	4I	4C	Severe Misfit	999	0	0	1	3	996
MLMV	Original	500	Moderate	4I	4C	Correct	999	0	0	1	1	998
MLMV	Collapsed	250	Moderate	4I	4C	Correct	987	0	0	11	24	965
MLMV	Collapsed	250	Moderate	4I	4C	Local Misfit	997	0	0	2	32	966
MLMV	Collapsed	250	Moderate	4I	4C	Severe Misfit	999	0	0	1	3	996
WLSMV	Original	250	Moderate	1I	1C	Correct	986	0	14	0	0	986
WLSMV	Original	250	Moderate	1I	1C	Local Misfit	986	0	14	0	0	986
WLSMV	Original	250	Moderate	1I	1C	Severe Misfit	986	0	14	0	0	986
WLSMV	Original	250	Moderate	1I	2C	Correct	986	0	14	0	0	986
WLSMV	Original	250	Moderate	1I	2C	Local Misfit	986	0	14	0	0	986
WLSMV	Original	250	Moderate	1I	2C	Severe Misfit	986	0	14	0	0	986
WLSMV	Original	250	Moderate	1I	4C	Correct	965	0	12	0	0	965
WLSMV	Original	250	Moderate	1I	4C	Local Misfit	965	0	12	0	1	964
WLSMV	Original	250	Moderate	1I	4C	Severe Misfit	965	0	12	0	0	965
WLSMV	Original	250	Moderate	2I	1C	Correct	969	0	31	0	0	969
WLSMV	Original	250	Moderate	2I	1C	Local Misfit	969	0	31	0	0	969
WLSMV	Original	250	Moderate	2I	1C	Severe Misfit	969	0	31	0	0	969
WLSMV	Original	250	Moderate	2I	2C	Correct	968	0	32	0	0	968
WLSMV	Original	250	Moderate	2I	2C	Local Misfit	968	0	32	0	0	968
WLSMV	Original	250	Moderate	2I	2C	Severe Misfit	968	0	32	0	0	968
WLSMV	Original	250	Moderate	2I	4C	Correct	930	0	29	0	5	925
WLSMV	Original	250	Moderate	2I	4C	Local Misfit	930	0	29	0	5	925
WLSMV	Original	250	Moderate	2I	4C	Severe Misfit	930	0	29	0	0	930
WLSMV	Original	250	Moderate	4I	1C	Correct	927	0	73	0	0	927
WLSMV	Original	250	Moderate	4I	1C	Local Misfit	927	0	73	0	0	927
WLSMV	Original	250	Moderate	4I	1C	Severe Misfit	927	0	73	0	0	927

Estimator	Handling	N	Loading	Scope	Pattern	Fitted model	Available R	Missing FI R	Empty-cat. R	Nonconv. R	Improper R	Admissible R
WLSMV	Original	250	Moderate	4I	2C	Correct	925	0	75	0	0	925
WLSMV	Original	250	Moderate	4I	2C	Local Misfit	925	0	75	0	0	925
WLSMV	Original	250	Moderate	4I	2C	Severe Misfit	925	0	75	0	0	925
WLSMV	Original	250	Moderate	4I	4C	Correct	819	0	68	0	75	766
WLSMV	Original	250	Moderate	4I	4C	Local Misfit	828	0	68	0	53	788
WLSMV	Original	250	Moderate	4I	4C	Severe Misfit	837	0	68	0	15	826
WLSMV	Original	250	High	1I	1C	Correct	986	0	14	0	2	984
WLSMV	Original	250	High	1I	1C	Local Misfit	986	0	14	0	306	680
WLSMV	Original	250	High	1I	1C	Severe Misfit	986	0	14	0	0	986
WLSMV	Original	250	High	1I	2C	Correct	984	0	16	0	2	982
WLSMV	Original	250	High	1I	2C	Local Misfit	984	0	16	0	312	672
WLSMV	Original	250	High	1I	2C	Severe Misfit	984	0	16	0	0	984
WLSMV	Original	250	High	1I	4C	Correct	965	0	16	0	7	958
WLSMV	Original	250	High	1I	4C	Local Misfit	965	0	16	0	400	565
WLSMV	Original	250	High	1I	4C	Severe Misfit	965	0	16	0	1	964
WLSMV	Original	250	High	2I	1C	Correct	966	0	34	0	1	965
WLSMV	Original	250	High	2I	1C	Local Misfit	966	0	34	0	307	659
WLSMV	Original	250	High	2I	1C	Severe Misfit	966	0	34	0	0	966
WLSMV	Original	250	High	2I	2C	Correct	964	0	36	0	2	962
WLSMV	Original	250	High	2I	2C	Local Misfit	964	0	36	0	332	632
WLSMV	Original	250	High	2I	2C	Severe Misfit	964	0	36	0	0	964
WLSMV	Original	250	High	2I	4C	Correct	927	0	34	0	11	916
WLSMV	Original	250	High	2I	4C	Local Misfit	927	0	34	0	495	432
WLSMV	Original	250	High	2I	4C	Severe Misfit	927	0	34	0	0	927
WLSMV	Original	250	High	4I	1C	Correct	927	0	73	0	1	926
WLSMV	Original	250	High	4I	1C	Local Misfit	927	0	73	0	330	597
WLSMV	Original	250	High	4I	1C	Severe Misfit	927	0	73	0	0	927
WLSMV	Original	250	High	4I	2C	Correct	923	0	77	0	1	922
WLSMV	Original	250	High	4I	2C	Local Misfit	923	0	77	0	377	546
WLSMV	Original	250	High	4I	2C	Severe Misfit	923	0	77	0	0	923
WLSMV	Original	250	High	4I	4C	Correct	862	0	73	0	133	729
WLSMV	Original	250	High	4I	4C	Local Misfit	862	0	73	0	486	376
WLSMV	Original	250	High	4I	4C	Severe Misfit	862	0	73	0	5	857
WLSMV	Original	500	Moderate	2I	4C	Correct	999	0	0	0	1	999
WLSMV	Original	500	Moderate	4I	4C	Correct	999	0	0	0	3	997
WLSMV	Collapsed	250	Moderate	4I	4C	Correct	993	0	0	2	35	963
WLSMV	Collapsed	500	Moderate	2I	4C	Correct	999	0	0	0	1	999

Note. A total of 1,000 replications were requested for each fitted model within each design cell. The table reports only conditions with Available R < 1,000 or at least one missing fit-index, empty-category, nonconvergence, or improper-solution flag; the remaining 895 fitted-model conditions yielded 1,000 available results with zero flags in these categories. Available R is the number of replications with a saved Mplus results record. Missing FI R denotes saved replications with at least one unavailable fit index; six such cases occurred, all involving unavailable RMSEA under MLR Severe Misfit. Empty-cat. R, Nonconv. R, and Improper R denote replications flagged for empty response categories, nonconvergence, and inadmissible solutions, respectively. Improper solutions included non-positive-definite residual or latent covariance matrices and related improper parameter solutions. Admissible R is the number of replications with complete CFI, TLI, RMSEA, and SRMR after excluding improper solutions and was used only in the sensitivity analysis. Diagnostic categories may overlap and should not be summed to reconstruct Available R. Handling = original five-category responses (Original) or deterministic category collapsing (Collapsed); 1I, 2I, and 4I = 1, 2, or 4 sparse items per factor; 1C, 2C, and 4C = the three marginal sparsity patterns in Table 1. CFI = comparative fit index; TLI = Tucker-Lewis index; RMSEA = root mean square error of approximation; SRMR = standardized root mean square residual; FI = fit index; MLR = robust maximum likelihood; MLMV = mean- and variance-adjusted maximum likelihood; WLSMV = weighted least squares mean and variance adjusted.

Table S2

Full Operating Characteristics Across All Scenarios and Decision Rules

Estimator	Category handling	N	Loading	Rule	False rejection	Local Misfit detection	Severe Misfit detection
MLR	Original	250	Moderate	CFI	0.078 [0.072, 0.084]	0.119 [0.112, 0.125]	0.644 [0.634, 0.654]
MLR	Original	250	Moderate	TLI	0.078 [0.072, 0.084]	0.116 [0.109, 0.122]	0.617 [0.607, 0.627]
MLR	Original	250	Moderate	RMSEA	0.062 [0.057, 0.066]	0.132 [0.125, 0.139]	0.681 [0.671, 0.690]
MLR	Original	250	Moderate	SRMR	0.093 [0.087, 0.099]	0.187 [0.179, 0.195]	0.798 [0.789, 0.806]
MLR	Original	250	Moderate	CFI-RMSEA AND	0.099 [0.092, 0.105]	0.176 [0.168, 0.184]	0.742 [0.733, 0.751]
MLR	Original	250	Moderate	2-of-3: CFI/RMSEA/SRMR	0.076 [0.071, 0.082]	0.143 [0.136, 0.151]	0.721 [0.712, 0.731]
MLR	Original	250	Moderate	CFI-RMSEA OR acceptance	0.041 [0.037, 0.045]	0.075 [0.070, 0.081]	0.583 [0.572, 0.593]
MLR	Original	500	Moderate	CFI	0.060 [0.056, 0.065]	0.201 [0.192, 0.209]	0.966 [0.963, 0.970]
MLR	Original	500	Moderate	TLI	0.060 [0.056, 0.065]	0.197 [0.189, 0.205]	0.964 [0.960, 0.968]
MLR	Original	500	Moderate	RMSEA	0.055 [0.050, 0.060]	0.276 [0.267, 0.286]	0.936 [0.931, 0.941]
MLR	Original	500	Moderate	SRMR	0.092 [0.086, 0.098]	0.328 [0.318, 0.337]	0.987 [0.985, 0.989]
MLR	Original	500	Moderate	CFI-RMSEA AND	0.083 [0.077, 0.089]	0.312 [0.302, 0.321]	0.972 [0.969, 0.976]
MLR	Original	500	Moderate	2-of-3: CFI/RMSEA/SRMR	0.064 [0.059, 0.070]	0.268 [0.259, 0.277]	0.971 [0.967, 0.974]
MLR	Original	500	Moderate	CFI-RMSEA OR acceptance	0.032 [0.029, 0.036]	0.165 [0.158, 0.173]	0.931 [0.925, 0.936]
MLR	Original	1,000	Moderate	CFI	0.059 [0.054, 0.064]	0.533 [0.522, 0.543]	0.997 [0.995, 0.998]
MLR	Original	1,000	Moderate	TLI	0.059 [0.054, 0.064]	0.526 [0.516, 0.537]	0.997 [0.995, 0.998]
MLR	Original	1,000	Moderate	RMSEA	0.052 [0.048, 0.057]	0.614 [0.604, 0.624]	0.986 [0.984, 0.989]
MLR	Original	1,000	Moderate	SRMR	0.105 [0.098, 0.111]	0.697 [0.687, 0.706]	1.000 [0.999, 1.000]
MLR	Original	1,000	Moderate	CFI-RMSEA AND	0.082 [0.076, 0.087]	0.658 [0.649, 0.668]	0.997 [0.995, 0.998]
MLR	Original	1,000	Moderate	2-of-3: CFI/RMSEA/SRMR	0.063 [0.058, 0.068]	0.627 [0.617, 0.637]	0.997 [0.995, 0.998]
MLR	Original	1,000	Moderate	CFI-RMSEA OR acceptance	0.029 [0.026, 0.033]	0.488 [0.477, 0.498]	0.986 [0.984, 0.989]
MLR	Original	250	High	CFI	0.156 [0.149, 0.164]	0.389 [0.379, 0.400]	1.000 [1.000, 1.000]
MLR	Original	250	High	TLI	0.156 [0.149, 0.164]	0.380 [0.370, 0.390]	1.000 [1.000, 1.000]
MLR	Original	250	High	RMSEA	0.129 [0.122, 0.136]	0.710 [0.701, 0.720]	1.000 [1.000, 1.000]
MLR	Original	250	High	SRMR	0.199 [0.191, 0.208]	0.641 [0.631, 0.651]	1.000 [1.000, 1.000]
MLR	Original	250	High	CFI-RMSEA AND	0.170 [0.162, 0.178]	0.733 [0.724, 0.742]	1.000 [1.000, 1.000]
MLR	Original	250	High	2-of-3: CFI/RMSEA/SRMR	0.160 [0.153, 0.168]	0.604 [0.594, 0.614]	1.000 [1.000, 1.000]
MLR	Original	250	High	CFI-RMSEA OR acceptance	0.115 [0.109, 0.122]	0.366 [0.356, 0.376]	1.000 [1.000, 1.000]
MLR	Original	500	High	CFI	0.199 [0.191, 0.207]	0.813 [0.805, 0.821]	1.000 [1.000, 1.000]
MLR	Original	500	High	TLI	0.199 [0.191, 0.207]	0.804 [0.795, 0.812]	1.000 [1.000, 1.000]
MLR	Original	500	High	RMSEA	0.174 [0.166, 0.182]	0.985 [0.982, 0.987]	1.000 [1.000, 1.000]
MLR	Original	500	High	SRMR	0.220 [0.212, 0.229]	0.975 [0.971, 0.978]	1.000 [1.000, 1.000]
MLR	Original	500	High	CFI-RMSEA AND	0.210 [0.201, 0.218]	0.988 [0.986, 0.990]	1.000 [1.000, 1.000]
MLR	Original	500	High	2-of-3: CFI/RMSEA/SRMR	0.203 [0.194, 0.211]	0.971 [0.968, 0.975]	1.000 [1.000, 1.000]
MLR	Original	500	High	CFI-RMSEA OR acceptance	0.164 [0.156, 0.172]	0.810 [0.802, 0.818]	1.000 [1.000, 1.000]
MLR	Original	1,000	High	CFI	0.221 [0.212, 0.230]	0.996 [0.995, 0.997]	1.000 [1.000, 1.000]
MLR	Original	1,000	High	TLI	0.221 [0.212, 0.230]	0.996 [0.994, 0.997]	1.000 [1.000, 1.000]
MLR	Original	1,000	High	RMSEA	0.217 [0.209, 0.226]	1.000 [1.000, 1.000]	1.000 [1.000, 1.000]
MLR	Original	1,000	High	SRMR	0.224 [0.215, 0.233]	1.000 [1.000, 1.000]	1.000 [1.000, 1.000]
MLR	Original	1,000	High	CFI-RMSEA AND	0.223 [0.215, 0.232]	1.000 [1.000, 1.000]	1.000 [1.000, 1.000]
MLR	Original	1,000	High	2-of-3: CFI/RMSEA/SRMR	0.222 [0.213, 0.230]	1.000 [1.000, 1.000]	1.000 [1.000, 1.000]
MLR	Original	1,000	High	CFI-RMSEA OR acceptance	0.215 [0.206, 0.223]	0.996 [0.995, 0.997]	1.000 [1.000, 1.000]
MLR	Collapsed	250	Moderate	CFI	0.064 [0.059, 0.069]	0.115 [0.108, 0.122]	0.672 [0.662, 0.682]
MLR	Collapsed	250	Moderate	TLI	0.064 [0.059, 0.069]	0.110 [0.104, 0.117]	0.651 [0.641, 0.660]
MLR	Collapsed	250	Moderate	RMSEA	0.051 [0.047, 0.056]	0.125 [0.118, 0.132]	0.672 [0.662, 0.682]
MLR	Collapsed	250	Moderate	SRMR	0.074 [0.068, 0.079]	0.174 [0.166, 0.182]	0.788 [0.779, 0.796]
MLR	Collapsed	250	Moderate	CFI-RMSEA AND	0.084 [0.078, 0.090]	0.165 [0.158, 0.173]	0.737 [0.728, 0.746]
MLR	Collapsed	250	Moderate	2-of-3: CFI/RMSEA/SRMR	0.060 [0.055, 0.065]	0.135 [0.128, 0.142]	0.720 [0.711, 0.729]
MLR	Collapsed	250	Moderate	CFI-RMSEA OR acceptance	0.031 [0.027, 0.034]	0.075 [0.069, 0.080]	0.607 [0.597, 0.617]
MLR	Collapsed	500	Moderate	CFI	0.056 [0.051, 0.061]	0.217 [0.208, 0.225]	0.965 [0.961, 0.969]
MLR	Collapsed	500	Moderate	TLI	0.056 [0.051, 0.061]	0.212 [0.203, 0.220]	0.963 [0.959, 0.967]
MLR	Collapsed	500	Moderate	RMSEA	0.051 [0.046, 0.055]	0.266 [0.257, 0.275]	0.927 [0.922, 0.932]
MLR	Collapsed	500	Moderate	SRMR	0.078 [0.073, 0.084]	0.325 [0.316, 0.335]	0.981 [0.978, 0.984]
MLR	Collapsed	500	Moderate	CFI-RMSEA AND	0.076 [0.071, 0.082]	0.304 [0.295, 0.314]	0.968 [0.964, 0.971]
MLR	Collapsed	500	Moderate	2-of-3: CFI/RMSEA/SRMR	0.060 [0.055, 0.065]	0.269 [0.260, 0.278]	0.967 [0.963, 0.970]
MLR	Collapsed	500	Moderate	CFI-RMSEA OR acceptance	0.030 [0.027, 0.034]	0.179 [0.171, 0.186]	0.924 [0.919, 0.929]
MLR	Collapsed	1,000	Moderate	CFI	0.053 [0.048, 0.058]	0.541 [0.531, 0.551]	0.997 [0.995, 0.998]
MLR	Collapsed	1,000	Moderate	TLI	0.053 [0.048, 0.058]	0.535 [0.525, 0.546]	0.997 [0.995, 0.998]

MLR	Collapsed	1,000	Moderate	RMSEA	0.052 [0.047, 0.057]	0.598 [0.588, 0.608]	0.985 [0.982, 0.988]
MLR	Collapsed	1,000	Moderate	SRMR	0.080 [0.075, 0.086]	0.696 [0.687, 0.706]	0.999 [0.999, 1.000]
MLR	Collapsed	1,000	Moderate	CFI-RMSEA AND	0.075 [0.070, 0.081]	0.639 [0.629, 0.649]	0.997 [0.995, 0.998]
MLR	Collapsed	1,000	Moderate	2-of-3: CFI/RMSEA/SRMR	0.058 [0.054, 0.063]	0.618 [0.608, 0.628]	0.997 [0.995, 0.998]
MLR	Collapsed	1,000	Moderate	CFI-RMSEA OR acceptance	0.030 [0.026, 0.033]	0.500 [0.489, 0.510]	0.985 [0.982, 0.988]
MLR	Collapsed	250	High	CFI	0.101 [0.095, 0.108]	0.576 [0.566, 0.587]	1.000 [1.000, 1.000]
MLR	Collapsed	250	High	TLI	0.101 [0.095, 0.108]	0.567 [0.557, 0.578]	1.000 [1.000, 1.000]
MLR	Collapsed	250	High	RMSEA	0.080 [0.074, 0.085]	0.753 [0.744, 0.762]	1.000 [1.000, 1.000]
MLR	Collapsed	250	High	SRMR	0.165 [0.157, 0.173]	0.757 [0.748, 0.766]	1.000 [1.000, 1.000]
MLR	Collapsed	250	High	CFI-RMSEA AND	0.120 [0.113, 0.126]	0.785 [0.777, 0.794]	1.000 [1.000, 1.000]
MLR	Collapsed	250	High	2-of-3: CFI/RMSEA/SRMR	0.105 [0.099, 0.112]	0.710 [0.700, 0.719]	1.000 [1.000, 1.000]
MLR	Collapsed	250	High	CFI-RMSEA OR acceptance	0.061 [0.056, 0.066]	0.544 [0.534, 0.555]	1.000 [1.000, 1.000]
MLR	Collapsed	500	High	CFI	0.144 [0.136, 0.151]	0.960 [0.956, 0.964]	1.000 [1.000, 1.000]
MLR	Collapsed	500	High	TLI	0.144 [0.136, 0.151]	0.958 [0.954, 0.962]	1.000 [1.000, 1.000]
MLR	Collapsed	500	High	RMSEA	0.118 [0.112, 0.125]	0.989 [0.987, 0.991]	1.000 [1.000, 1.000]
MLR	Collapsed	500	High	SRMR	0.193 [0.185, 0.201]	0.994 [0.992, 0.996]	1.000 [1.000, 1.000]
MLR	Collapsed	500	High	CFI-RMSEA AND	0.161 [0.153, 0.168]	0.994 [0.992, 0.995]	1.000 [1.000, 1.000]
MLR	Collapsed	500	High	2-of-3: CFI/RMSEA/SRMR	0.149 [0.142, 0.156]	0.992 [0.990, 0.994]	1.000 [1.000, 1.000]
MLR	Collapsed	500	High	CFI-RMSEA OR acceptance	0.101 [0.095, 0.107]	0.956 [0.951, 0.960]	1.000 [1.000, 1.000]
MLR	Collapsed	1,000	High	CFI	0.200 [0.192, 0.208]	1.000 [1.000, 1.000]	1.000 [1.000, 1.000]
MLR	Collapsed	1,000	High	TLI	0.200 [0.192, 0.208]	1.000 [1.000, 1.000]	1.000 [1.000, 1.000]
MLR	Collapsed	1,000	High	RMSEA	0.195 [0.187, 0.204]	1.000 [1.000, 1.000]	1.000 [1.000, 1.000]
MLR	Collapsed	1,000	High	SRMR	0.218 [0.209, 0.226]	1.000 [1.000, 1.000]	1.000 [1.000, 1.000]
MLR	Collapsed	1,000	High	CFI-RMSEA AND	0.215 [0.207, 0.224]	1.000 [1.000, 1.000]	1.000 [1.000, 1.000]
MLR	Collapsed	1,000	High	2-of-3: CFI/RMSEA/SRMR	0.206 [0.198, 0.214]	1.000 [1.000, 1.000]	1.000 [1.000, 1.000]
MLR	Collapsed	1,000	High	CFI-RMSEA OR acceptance	0.180 [0.172, 0.188]	1.000 [1.000, 1.000]	1.000 [1.000, 1.000]
MLMV	Original	250	Moderate	CFI	0.062 [0.057, 0.067]	0.116 [0.109, 0.122]	0.690 [0.681, 0.700]
MLMV	Original	250	Moderate	TLI	0.062 [0.057, 0.067]	0.112 [0.106, 0.119]	0.669 [0.659, 0.678]
MLMV	Original	250	Moderate	RMSEA	0.050 [0.046, 0.055]	0.136 [0.129, 0.143]	0.684 [0.675, 0.694]
MLMV	Original	250	Moderate	SRMR	0.093 [0.087, 0.099]	0.186 [0.178, 0.194]	0.797 [0.789, 0.805]
MLMV	Original	250	Moderate	CFI-RMSEA AND	0.094 [0.088, 0.100]	0.191 [0.183, 0.199]	0.775 [0.766, 0.784]
MLMV	Original	250	Moderate	2-of-3: CFI/RMSEA/SRMR	0.065 [0.060, 0.070]	0.143 [0.136, 0.151]	0.744 [0.735, 0.753]
MLMV	Original	250	Moderate	CFI-RMSEA OR acceptance	0.018 [0.016, 0.021]	0.061 [0.056, 0.066]	0.599 [0.589, 0.609]
MLMV	Original	500	Moderate	CFI	0.053 [0.048, 0.057]	0.200 [0.192, 0.208]	0.963 [0.959, 0.967]
MLMV	Original	500	Moderate	TLI	0.053 [0.048, 0.057]	0.195 [0.186, 0.203]	0.961 [0.957, 0.965]
MLMV	Original	500	Moderate	RMSEA	0.051 [0.046, 0.056]	0.279 [0.270, 0.288]	0.916 [0.911, 0.922]
MLMV	Original	500	Moderate	SRMR	0.092 [0.086, 0.098]	0.328 [0.318, 0.337]	0.987 [0.985, 0.989]
MLMV	Original	500	Moderate	CFI-RMSEA AND	0.084 [0.078, 0.090]	0.330 [0.320, 0.340]	0.969 [0.965, 0.972]
MLMV	Original	500	Moderate	2-of-3: CFI/RMSEA/SRMR	0.056 [0.051, 0.061]	0.266 [0.257, 0.275]	0.967 [0.963, 0.970]
MLMV	Original	500	Moderate	CFI-RMSEA OR acceptance	0.020 [0.017, 0.022]	0.148 [0.141, 0.156]	0.911 [0.905, 0.917]
MLMV	Original	1,000	Moderate	CFI	0.056 [0.051, 0.060]	0.533 [0.522, 0.543]	0.996 [0.995, 0.998]
MLMV	Original	1,000	Moderate	TLI	0.056 [0.051, 0.060]	0.526 [0.515, 0.536]	0.996 [0.995, 0.998]
MLMV	Original	1,000	Moderate	RMSEA	0.051 [0.047, 0.056]	0.614 [0.603, 0.624]	0.981 [0.978, 0.984]
MLMV	Original	1,000	Moderate	SRMR	0.105 [0.098, 0.111]	0.697 [0.687, 0.706]	1.000 [0.999, 1.000]
MLMV	Original	1,000	Moderate	CFI-RMSEA AND	0.084 [0.078, 0.090]	0.671 [0.661, 0.680]	0.996 [0.995, 0.998]
MLMV	Original	1,000	Moderate	2-of-3: CFI/RMSEA/SRMR	0.060 [0.055, 0.065]	0.630 [0.620, 0.640]	0.996 [0.995, 0.998]
MLMV	Original	1,000	Moderate	CFI-RMSEA OR acceptance	0.023 [0.020, 0.026]	0.475 [0.465, 0.486]	0.981 [0.978, 0.984]
MLMV	Original	250	High	CFI	0.150 [0.142, 0.157]	0.376 [0.366, 0.386]	1.000 [1.000, 1.000]
MLMV	Original	250	High	TLI	0.150 [0.142, 0.157]	0.367 [0.357, 0.377]	1.000 [1.000, 1.000]
MLMV	Original	250	High	RMSEA	0.094 [0.088, 0.100]	0.720 [0.711, 0.730]	0.999 [0.999, 1.000]
MLMV	Original	250	High	SRMR	0.199 [0.191, 0.208]	0.641 [0.631, 0.651]	1.000 [1.000, 1.000]
MLMV	Original	250	High	CFI-RMSEA AND	0.167 [0.159, 0.175]	0.789 [0.781, 0.798]	1.000 [1.000, 1.000]
MLMV	Original	250	High	2-of-3: CFI/RMSEA/SRMR	0.151 [0.143, 0.158]	0.613 [0.603, 0.623]	1.000 [1.000, 1.000]
MLMV	Original	250	High	CFI-RMSEA OR acceptance	0.076 [0.071, 0.082]	0.307 [0.297, 0.317]	0.999 [0.999, 1.000]
MLMV	Original	500	High	CFI	0.197 [0.189, 0.205]	0.740 [0.730, 0.749]	1.000 [1.000, 1.000]
MLMV	Original	500	High	TLI	0.197 [0.189, 0.205]	0.727 [0.718, 0.737]	1.000 [1.000, 1.000]
MLMV	Original	500	High	RMSEA	0.147 [0.140, 0.155]	0.978 [0.975, 0.981]	1.000 [1.000, 1.000]
MLMV	Original	500	High	SRMR	0.220 [0.212, 0.229]	0.975 [0.971, 0.978]	1.000 [1.000, 1.000]
MLMV	Original	500	High	CFI-RMSEA AND	0.208 [0.200, 0.216]	0.991 [0.989, 0.993]	1.000 [1.000, 1.000]
MLMV	Original	500	High	2-of-3: CFI/RMSEA/SRMR	0.199 [0.191, 0.207]	0.972 [0.969, 0.976]	1.000 [1.000, 1.000]
MLMV	Original	500	High	CFI-RMSEA OR acceptance	0.136 [0.129, 0.143]	0.727 [0.717, 0.736]	1.000 [1.000, 1.000]
MLMV	Original	1,000	High	CFI	0.221 [0.212, 0.230]	0.987 [0.985, 0.990]	1.000 [1.000, 1.000]
MLMV	Original	1,000	High	TLI	0.221 [0.212, 0.230]	0.986 [0.984, 0.989]	1.000 [1.000, 1.000]
MLMV	Original	1,000	High	RMSEA	0.208 [0.199, 0.216]	1.000 [1.000, 1.000]	1.000 [1.000, 1.000]
MLMV	Original	1,000	High	SRMR	0.224 [0.215, 0.233]	1.000 [1.000, 1.000]	1.000 [1.000, 1.000]
MLMV	Original	1,000	High	CFI-RMSEA AND	0.225 [0.216, 0.234]	1.000 [1.000, 1.000]	1.000 [1.000, 1.000]

MLMV	Original	1,000	High	2-of-3: CFI/RMSEA/SRMR	0.222 [0.213, 0.230]	1.000 [1.000, 1.000]	1.000 [1.000, 1.000]
MLMV	Original	1,000	High	CFI-RMSEA OR acceptance	0.203 [0.195, 0.212]	0.987 [0.985, 0.990]	1.000 [1.000, 1.000]
MLMV	Collapsed	250	Moderate	CFI	0.056 [0.051, 0.060]	0.114 [0.108, 0.121]	0.695 [0.685, 0.704]
MLMV	Collapsed	250	Moderate	TLI	0.056 [0.051, 0.060]	0.111 [0.104, 0.117]	0.673 [0.663, 0.683]
MLMV	Collapsed	250	Moderate	RMSEA	0.051 [0.047, 0.056]	0.130 [0.123, 0.137]	0.678 [0.669, 0.688]
MLMV	Collapsed	250	Moderate	SRMR	0.074 [0.068, 0.079]	0.174 [0.166, 0.182]	0.788 [0.779, 0.796]
MLMV	Collapsed	250	Moderate	CFI-RMSEA AND	0.082 [0.076, 0.087]	0.171 [0.163, 0.179]	0.758 [0.749, 0.766]
MLMV	Collapsed	250	Moderate	2-of-3: CFI/RMSEA/SRMR	0.058 [0.053, 0.062]	0.135 [0.128, 0.142]	0.735 [0.726, 0.744]
MLMV	Collapsed	250	Moderate	CFI-RMSEA OR acceptance	0.025 [0.022, 0.028]	0.073 [0.068, 0.078]	0.615 [0.605, 0.625]
MLMV	Collapsed	500	Moderate	CFI	0.053 [0.048, 0.057]	0.219 [0.210, 0.228]	0.964 [0.960, 0.968]
MLMV	Collapsed	500	Moderate	TLI	0.053 [0.048, 0.057]	0.214 [0.206, 0.222]	0.962 [0.958, 0.966]
MLMV	Collapsed	500	Moderate	RMSEA	0.051 [0.047, 0.056]	0.268 [0.259, 0.277]	0.918 [0.912, 0.923]
MLMV	Collapsed	500	Moderate	SRMR	0.078 [0.073, 0.084]	0.325 [0.316, 0.335]	0.981 [0.978, 0.984]
MLMV	Collapsed	500	Moderate	CFI-RMSEA AND	0.077 [0.072, 0.083]	0.312 [0.302, 0.321]	0.967 [0.964, 0.971]
MLMV	Collapsed	500	Moderate	2-of-3: CFI/RMSEA/SRMR	0.057 [0.052, 0.062]	0.270 [0.261, 0.280]	0.966 [0.962, 0.969]
MLMV	Collapsed	500	Moderate	CFI-RMSEA OR acceptance	0.026 [0.023, 0.030]	0.176 [0.168, 0.184]	0.915 [0.909, 0.920]
MLMV	Collapsed	1,000	Moderate	CFI	0.052 [0.048, 0.057]	0.537 [0.527, 0.548]	0.997 [0.995, 0.998]
MLMV	Collapsed	1,000	Moderate	TLI	0.052 [0.048, 0.057]	0.531 [0.521, 0.542]	0.996 [0.995, 0.998]
MLMV	Collapsed	1,000	Moderate	RMSEA	0.050 [0.046, 0.055]	0.594 [0.584, 0.604]	0.982 [0.979, 0.985]
MLMV	Collapsed	1,000	Moderate	SRMR	0.080 [0.075, 0.086]	0.696 [0.687, 0.706]	0.999 [0.999, 1.000]
MLMV	Collapsed	1,000	Moderate	CFI-RMSEA AND	0.075 [0.070, 0.081]	0.641 [0.631, 0.651]	0.997 [0.995, 0.998]
MLMV	Collapsed	1,000	Moderate	2-of-3: CFI/RMSEA/SRMR	0.058 [0.053, 0.062]	0.618 [0.608, 0.628]	0.997 [0.995, 0.998]
MLMV	Collapsed	1,000	Moderate	CFI-RMSEA OR acceptance	0.027 [0.024, 0.030]	0.490 [0.480, 0.500]	0.982 [0.979, 0.985]
MLMV	Collapsed	250	High	CFI	0.105 [0.099, 0.112]	0.556 [0.545, 0.566]	1.000 [1.000, 1.000]
MLMV	Collapsed	250	High	TLI	0.105 [0.099, 0.112]	0.547 [0.536, 0.557]	1.000 [1.000, 1.000]
MLMV	Collapsed	250	High	RMSEA	0.068 [0.063, 0.073]	0.748 [0.739, 0.757]	1.000 [1.000, 1.000]
MLMV	Collapsed	250	High	SRMR	0.165 [0.157, 0.173]	0.757 [0.748, 0.766]	1.000 [1.000, 1.000]
MLMV	Collapsed	250	High	CFI-RMSEA AND	0.125 [0.118, 0.132]	0.797 [0.789, 0.806]	1.000 [1.000, 1.000]
MLMV	Collapsed	250	High	2-of-3: CFI/RMSEA/SRMR	0.106 [0.100, 0.113]	0.712 [0.703, 0.722]	1.000 [1.000, 1.000]
MLMV	Collapsed	250	High	CFI-RMSEA OR acceptance	0.048 [0.044, 0.053]	0.506 [0.496, 0.516]	1.000 [1.000, 1.000]
MLMV	Collapsed	500	High	CFI	0.147 [0.139, 0.154]	0.948 [0.943, 0.952]	1.000 [1.000, 1.000]
MLMV	Collapsed	500	High	TLI	0.147 [0.139, 0.154]	0.945 [0.941, 0.950]	1.000 [1.000, 1.000]
MLMV	Collapsed	500	High	RMSEA	0.110 [0.104, 0.117]	0.986 [0.984, 0.989]	1.000 [1.000, 1.000]
MLMV	Collapsed	500	High	SRMR	0.193 [0.185, 0.201]	0.994 [0.992, 0.996]	1.000 [1.000, 1.000]
MLMV	Collapsed	500	High	CFI-RMSEA AND	0.163 [0.155, 0.171]	0.994 [0.993, 0.996]	1.000 [1.000, 1.000]
MLMV	Collapsed	500	High	2-of-3: CFI/RMSEA/SRMR	0.150 [0.143, 0.157]	0.992 [0.990, 0.994]	1.000 [1.000, 1.000]
MLMV	Collapsed	500	High	CFI-RMSEA OR acceptance	0.094 [0.088, 0.100]	0.940 [0.935, 0.945]	1.000 [1.000, 1.000]
MLMV	Collapsed	1,000	High	CFI	0.201 [0.193, 0.209]	1.000 [1.000, 1.000]	1.000 [1.000, 1.000]
MLMV	Collapsed	1,000	High	TLI	0.201 [0.193, 0.209]	1.000 [1.000, 1.000]	1.000 [1.000, 1.000]
MLMV	Collapsed	1,000	High	RMSEA	0.190 [0.182, 0.198]	1.000 [1.000, 1.000]	1.000 [1.000, 1.000]
MLMV	Collapsed	1,000	High	SRMR	0.218 [0.209, 0.226]	1.000 [1.000, 1.000]	1.000 [1.000, 1.000]
MLMV	Collapsed	1,000	High	CFI-RMSEA AND	0.216 [0.208, 0.225]	1.000 [1.000, 1.000]	1.000 [1.000, 1.000]
MLMV	Collapsed	1,000	High	2-of-3: CFI/RMSEA/SRMR	0.206 [0.198, 0.215]	1.000 [1.000, 1.000]	1.000 [1.000, 1.000]
MLMV	Collapsed	1,000	High	CFI-RMSEA OR acceptance	0.175 [0.167, 0.183]	1.000 [1.000, 1.000]	1.000 [1.000, 1.000]
WLSMV	Original	250	Moderate	CFI	0.054 [0.049, 0.058]	0.065 [0.060, 0.070]	0.375 [0.365, 0.386]
WLSMV	Original	250	Moderate	TLI	0.054 [0.049, 0.058]	0.064 [0.058, 0.069]	0.348 [0.337, 0.358]
WLSMV	Original	250	Moderate	RMSEA	0.045 [0.041, 0.050]	0.075 [0.070, 0.081]	0.582 [0.572, 0.593]
WLSMV	Original	250	Moderate	SRMR	0.166 [0.158, 0.174]	0.182 [0.174, 0.190]	0.261 [0.252, 0.270]
WLSMV	Original	250	Moderate	CFI-RMSEA AND	0.058 [0.053, 0.063]	0.092 [0.086, 0.099]	0.619 [0.609, 0.629]
WLSMV	Original	250	Moderate	2-of-3: CFI/RMSEA/SRMR	0.054 [0.049, 0.059]	0.067 [0.062, 0.072]	0.387 [0.377, 0.397]
WLSMV	Original	250	Moderate	CFI-RMSEA OR acceptance	0.041 [0.037, 0.045]	0.048 [0.043, 0.052]	0.339 [0.329, 0.349]
WLSMV	Original	500	Moderate	CFI	0.061 [0.056, 0.066]	0.266 [0.257, 0.275]	0.981 [0.978, 0.984]
WLSMV	Original	500	Moderate	TLI	0.061 [0.056, 0.066]	0.260 [0.251, 0.269]	0.978 [0.975, 0.981]
WLSMV	Original	500	Moderate	RMSEA	0.053 [0.048, 0.057]	0.335 [0.325, 0.345]	0.954 [0.949, 0.958]
WLSMV	Original	500	Moderate	SRMR	0.224 [0.215, 0.233]	0.261 [0.252, 0.271]	0.885 [0.878, 0.891]
WLSMV	Original	500	Moderate	CFI-RMSEA AND	0.083 [0.077, 0.089]	0.376 [0.366, 0.386]	0.984 [0.981, 0.986]
WLSMV	Original	500	Moderate	2-of-3: CFI/RMSEA/SRMR	0.063 [0.058, 0.068]	0.271 [0.261, 0.280]	0.981 [0.978, 0.984]
WLSMV	Original	500	Moderate	CFI-RMSEA OR acceptance	0.030 [0.027, 0.034]	0.225 [0.216, 0.233]	0.951 [0.946, 0.955]
WLSMV	Original	1,000	Moderate	CFI	0.056 [0.051, 0.061]	0.662 [0.652, 0.672]	0.998 [0.997, 0.999]
WLSMV	Original	1,000	Moderate	TLI	0.056 [0.051, 0.061]	0.656 [0.647, 0.666]	0.998 [0.997, 0.999]
WLSMV	Original	1,000	Moderate	RMSEA	0.050 [0.046, 0.055]	0.700 [0.691, 0.710]	0.993 [0.992, 0.995]
WLSMV	Original	1,000	Moderate	SRMR	0.229 [0.221, 0.238]	0.327 [0.318, 0.337]	1.000 [1.000, 1.000]
WLSMV	Original	1,000	Moderate	CFI-RMSEA AND	0.076 [0.071, 0.082]	0.740 [0.731, 0.749]	0.998 [0.997, 0.999]
WLSMV	Original	1,000	Moderate	2-of-3: CFI/RMSEA/SRMR	0.057 [0.053, 0.062]	0.665 [0.655, 0.674]	0.998 [0.997, 0.999]
WLSMV	Original	1,000	Moderate	CFI-RMSEA OR acceptance	0.030 [0.027, 0.034]	0.622 [0.612, 0.632]	0.993 [0.992, 0.995]
WLSMV	Original	250	High	CFI	0.054 [0.049, 0.059]	0.704 [0.694, 0.714]	1.000 [1.000, 1.000]

WLSMV	Original	250	High	TLI	0.054 [0.049, 0.059]	0.697 [0.688, 0.707]	1.000 [1.000, 1.000]
WLSMV	Original	250	High	RMSEA	0.050 [0.045, 0.054]	0.779 [0.771, 0.788]	1.000 [1.000, 1.000]
WLSMV	Original	250	High	SRMR	0.198 [0.190, 0.207]	0.303 [0.293, 0.312]	1.000 [1.000, 1.000]
WLSMV	Original	250	High	CFI-RMSEA AND	0.075 [0.069, 0.080]	0.810 [0.802, 0.818]	1.000 [1.000, 1.000]
WLSMV	Original	250	High	2-of-3: CFI/RMSEA/SRMR	0.057 [0.052, 0.062]	0.710 [0.701, 0.720]	1.000 [1.000, 1.000]
WLSMV	Original	250	High	CFI-RMSEA OR acceptance	0.029 [0.026, 0.033]	0.673 [0.663, 0.683]	1.000 [1.000, 1.000]
WLSMV	Original	500	High	CFI	0.056 [0.052, 0.061]	0.982 [0.979, 0.984]	1.000 [1.000, 1.000]
WLSMV	Original	500	High	TLI	0.056 [0.052, 0.061]	0.981 [0.979, 0.984]	1.000 [1.000, 1.000]
WLSMV	Original	500	High	RMSEA	0.050 [0.046, 0.055]	0.956 [0.952, 0.960]	1.000 [1.000, 1.000]
WLSMV	Original	500	High	SRMR	0.223 [0.214, 0.231]	0.726 [0.716, 0.735]	1.000 [1.000, 1.000]
WLSMV	Original	500	High	CFI-RMSEA AND	0.073 [0.067, 0.078]	0.983 [0.980, 0.985]	1.000 [1.000, 1.000]
WLSMV	Original	500	High	2-of-3: CFI/RMSEA/SRMR	0.058 [0.053, 0.063]	0.982 [0.979, 0.985]	1.000 [1.000, 1.000]
WLSMV	Original	500	High	CFI-RMSEA OR acceptance	0.034 [0.030, 0.038]	0.955 [0.951, 0.959]	1.000 [1.000, 1.000]
WLSMV	Original	1,000	High	CFI	0.051 [0.047, 0.056]	0.999 [0.999, 1.000]	1.000 [1.000, 1.000]
WLSMV	Original	1,000	High	TLI	0.051 [0.047, 0.056]	0.999 [0.999, 1.000]	1.000 [1.000, 1.000]
WLSMV	Original	1,000	High	RMSEA	0.050 [0.045, 0.054]	0.996 [0.995, 0.997]	1.000 [1.000, 1.000]
WLSMV	Original	1,000	High	SRMR	0.234 [0.226, 0.243]	1.000 [1.000, 1.000]	1.000 [1.000, 1.000]
WLSMV	Original	1,000	High	CFI-RMSEA AND	0.068 [0.063, 0.073]	0.999 [0.999, 1.000]	1.000 [1.000, 1.000]
WLSMV	Original	1,000	High	2-of-3: CFI/RMSEA/SRMR	0.053 [0.048, 0.058]	0.999 [0.999, 1.000]	1.000 [1.000, 1.000]
WLSMV	Original	1,000	High	CFI-RMSEA OR acceptance	0.033 [0.029, 0.037]	0.996 [0.995, 0.997]	1.000 [1.000, 1.000]
WLSMV	Collapsed	250	Moderate	CFI	0.056 [0.051, 0.061]	0.129 [0.122, 0.136]	0.744 [0.735, 0.753]
WLSMV	Collapsed	250	Moderate	TLI	0.056 [0.051, 0.061]	0.125 [0.118, 0.132]	0.729 [0.720, 0.739]
WLSMV	Collapsed	250	Moderate	RMSEA	0.051 [0.046, 0.055]	0.149 [0.141, 0.156]	0.740 [0.731, 0.749]
WLSMV	Collapsed	250	Moderate	SRMR	0.216 [0.207, 0.224]	0.231 [0.222, 0.239]	0.356 [0.346, 0.365]
WLSMV	Collapsed	250	Moderate	CFI-RMSEA AND	0.078 [0.073, 0.084]	0.188 [0.180, 0.196]	0.806 [0.798, 0.814]
WLSMV	Collapsed	250	Moderate	2-of-3: CFI/RMSEA/SRMR	0.056 [0.052, 0.061]	0.127 [0.120, 0.134]	0.741 [0.732, 0.750]
WLSMV	Collapsed	250	Moderate	CFI-RMSEA OR acceptance	0.028 [0.025, 0.032]	0.089 [0.083, 0.095]	0.677 [0.668, 0.687]
WLSMV	Collapsed	500	Moderate	CFI	0.053 [0.048, 0.058]	0.266 [0.257, 0.275]	0.970 [0.966, 0.973]
WLSMV	Collapsed	500	Moderate	TLI	0.053 [0.048, 0.058]	0.260 [0.251, 0.269]	0.967 [0.963, 0.971]
WLSMV	Collapsed	500	Moderate	RMSEA	0.050 [0.046, 0.055]	0.325 [0.315, 0.334]	0.932 [0.927, 0.938]
WLSMV	Collapsed	500	Moderate	SRMR	0.218 [0.209, 0.226]	0.249 [0.240, 0.257]	0.779 [0.771, 0.788]
WLSMV	Collapsed	500	Moderate	CFI-RMSEA AND	0.075 [0.070, 0.080]	0.361 [0.351, 0.370]	0.973 [0.970, 0.976]
WLSMV	Collapsed	500	Moderate	2-of-3: CFI/RMSEA/SRMR	0.054 [0.050, 0.059]	0.268 [0.259, 0.277]	0.970 [0.966, 0.973]
WLSMV	Collapsed	500	Moderate	CFI-RMSEA OR acceptance	0.028 [0.025, 0.032]	0.230 [0.221, 0.239]	0.929 [0.924, 0.934]
WLSMV	Collapsed	1,000	Moderate	CFI	0.048 [0.043, 0.052]	0.632 [0.622, 0.642]	0.997 [0.996, 0.998]
WLSMV	Collapsed	1,000	Moderate	TLI	0.048 [0.043, 0.052]	0.626 [0.616, 0.636]	0.997 [0.996, 0.998]
WLSMV	Collapsed	1,000	Moderate	RMSEA	0.051 [0.046, 0.055]	0.668 [0.658, 0.678]	0.988 [0.986, 0.990]
WLSMV	Collapsed	1,000	Moderate	SRMR	0.218 [0.209, 0.226]	0.303 [0.293, 0.312]	1.000 [0.999, 1.000]
WLSMV	Collapsed	1,000	Moderate	CFI-RMSEA AND	0.069 [0.064, 0.074]	0.708 [0.699, 0.718]	0.997 [0.996, 0.998]
WLSMV	Collapsed	1,000	Moderate	2-of-3: CFI/RMSEA/SRMR	0.049 [0.044, 0.053]	0.633 [0.623, 0.643]	0.997 [0.996, 0.998]
WLSMV	Collapsed	1,000	Moderate	CFI-RMSEA OR acceptance	0.029 [0.026, 0.033]	0.592 [0.582, 0.602]	0.988 [0.986, 0.990]
WLSMV	Collapsed	250	High	CFI	0.055 [0.050, 0.059]	0.753 [0.744, 0.762]	1.000 [1.000, 1.000]
WLSMV	Collapsed	250	High	TLI	0.055 [0.050, 0.059]	0.747 [0.738, 0.756]	1.000 [1.000, 1.000]
WLSMV	Collapsed	250	High	RMSEA	0.050 [0.046, 0.055]	0.768 [0.759, 0.777]	1.000 [0.999, 1.000]
WLSMV	Collapsed	250	High	SRMR	0.214 [0.206, 0.223]	0.299 [0.290, 0.309]	1.000 [1.000, 1.000]
WLSMV	Collapsed	250	High	CFI-RMSEA AND	0.072 [0.067, 0.077]	0.811 [0.803, 0.819]	1.000 [1.000, 1.000]
WLSMV	Collapsed	250	High	2-of-3: CFI/RMSEA/SRMR	0.056 [0.051, 0.060]	0.756 [0.747, 0.764]	1.000 [1.000, 1.000]
WLSMV	Collapsed	250	High	CFI-RMSEA OR acceptance	0.033 [0.029, 0.037]	0.710 [0.700, 0.719]	1.000 [0.999, 1.000]
WLSMV	Collapsed	500	High	CFI	0.054 [0.049, 0.059]	0.976 [0.973, 0.979]	1.000 [1.000, 1.000]
WLSMV	Collapsed	500	High	TLI	0.054 [0.049, 0.059]	0.976 [0.972, 0.979]	1.000 [1.000, 1.000]
WLSMV	Collapsed	500	High	RMSEA	0.050 [0.046, 0.055]	0.941 [0.936, 0.946]	1.000 [1.000, 1.000]
WLSMV	Collapsed	500	High	SRMR	0.219 [0.211, 0.228]	0.603 [0.593, 0.613]	1.000 [1.000, 1.000]
WLSMV	Collapsed	500	High	CFI-RMSEA AND	0.070 [0.065, 0.075]	0.977 [0.974, 0.980]	1.000 [1.000, 1.000]
WLSMV	Collapsed	500	High	2-of-3: CFI/RMSEA/SRMR	0.055 [0.050, 0.059]	0.976 [0.973, 0.979]	1.000 [1.000, 1.000]
WLSMV	Collapsed	500	High	CFI-RMSEA OR acceptance	0.034 [0.031, 0.038]	0.941 [0.936, 0.945]	1.000 [1.000, 1.000]
WLSMV	Collapsed	1,000	High	CFI	0.051 [0.046, 0.055]	0.999 [0.998, 0.999]	1.000 [1.000, 1.000]
WLSMV	Collapsed	1,000	High	TLI	0.051 [0.046, 0.055]	0.999 [0.998, 0.999]	1.000 [1.000, 1.000]
WLSMV	Collapsed	1,000	High	RMSEA	0.050 [0.046, 0.055]	0.992 [0.990, 0.994]	1.000 [1.000, 1.000]
WLSMV	Collapsed	1,000	High	SRMR	0.225 [0.217, 0.234]	0.998 [0.998, 0.999]	1.000 [1.000, 1.000]
WLSMV	Collapsed	1,000	High	CFI-RMSEA AND	0.069 [0.063, 0.074]	0.999 [0.998, 0.999]	1.000 [1.000, 1.000]
WLSMV	Collapsed	1,000	High	2-of-3: CFI/RMSEA/SRMR	0.053 [0.048, 0.057]	0.999 [0.998, 0.999]	1.000 [1.000, 1.000]
WLSMV	Collapsed	1,000	High	CFI-RMSEA OR acceptance	0.032 [0.029, 0.036]	0.992 [0.990, 0.994]	1.000 [1.000, 1.000]

Note. Values are scenario-level rates with approximate 95% Monte Carlo confidence intervals in brackets, computed using cutoffs calibrated at $\alpha = .05$ from Correct-model reference distributions. False rejection is the proportion of Correct-model replications flagged as misfitting. Local Misfit detection and Severe Misfit detection are the corresponding proportions flagged under the two misspecified fitted models. Local Misfit omitted the prespecified cross-loading; Severe Misfit combined the first two factors. Composite rules are empirical operating characteristics rather than nominal α-controlled tests. Abbreviations are defined in the main text.

Table S3

Sensitivity and Robustness Checks

Panel A. Calibration-level sensitivity for scenario-calibrated cutoffs

Index	α	Mean cutoff	Median cutoff	Minimum	Maximum	n
CFI	α = .01	0.953	0.970	0.820	0.997	36
CFI	α = .05	0.966	0.978	0.883	0.998	36
CFI	α = .10	0.973	0.983	0.908	0.998	36
TLI	α = .01	0.943	0.963	0.780	0.996	36
TLI	α = .05	0.959	0.973	0.857	0.998	36
TLI	α = .10	0.967	0.979	0.887	0.998	36
RMSEA	α = .01	0.032	0.029	0.019	0.055	36
RMSEA	α = .05	0.027	0.024	0.016	0.043	36
RMSEA	α = .10	0.024	0.021	0.014	0.039	36
SRMR	α = .01	0.041	0.038	0.024	0.073	36
SRMR	α = .05	0.038	0.036	0.023	0.065	36
SRMR	α = .10	0.036	0.035	0.022	0.063	36

Panel B. Extreme-sparsity trimming sensitivity

Quantity	Index or rule group	Sensitivity condition	Mean absolute change	Maximum absolute change	n
Scenario-level cutoff	CFI	Trim highest sparsity-severity tier (NCat = 4)	0.012	0.066	36
Scenario-level cutoff	TLI	Trim highest sparsity-severity tier (NCat = 4)	0.014	0.081	36
Scenario-level cutoff	RMSEA	Trim highest sparsity-severity tier (NCat = 4)	0.002	0.009	36
Scenario-level cutoff	SRMR	Trim highest sparsity-severity tier (NCat = 4)	0.005	0.018	36
False rejection	All single-index and composite decisions	Trim highest sparsity-severity tier (NCat = 4)	0.053	0.177	252
Local Misfit detection	All single-index and composite decisions	Trim highest sparsity-severity tier (NCat = 4)	0.095	0.537	252
Severe Misfit detection	All single-index and composite decisions	Trim highest sparsity-severity tier (NCat = 4)	0.042	0.652	252

Panel C. Realized false rejection under the Correct condition at α = .05

Index or rule	Mean false rejection	Median	Minimum	Maximum	Mean absolute deviation	Maximum absolute deviation	n
SRMR	0.169	0.199	0.074	0.234	0.119	0.184	36
CFI-RMSEA AND	0.113	0.083	0.058	0.225	0.063	0.175	36
2-of-3: CFI/RMSEA/SRMR	0.097	0.060	0.049	0.222	0.047	0.172	36
CFI	0.094	0.058	0.048	0.221	0.044	0.171	36
TLI	0.094	0.058	0.048	0.221	0.044	0.171	36
RMSEA	0.082	0.051	0.045	0.217	0.032	0.167	36
CFI-RMSEA OR acceptance	0.063	0.033	0.018	0.215	0.040	0.165	36

Note. Panel A summarizes scenario-level cutoffs at alternative calibration levels. Scenario-level cutoffs are unweighted means across the nine sparsity cells within each Estimator × Category Handling × Sample Size × Loading scenario; values summarize 36 scenarios. Panel B summarizes the absolute change after excluding the highest sparsity-severity tier (NCat = 4) and recomputing scenario-level cutoffs and operating characteristics. Operating-characteristic changes are summarized across all single-index and composite decisions. Panel C summarizes realized false rejection under the Correct condition for each single-index or composite decision at the primary calibration level, α = .05. Mean absolute deviation in Panel C refers to absolute deviation from the nominal .05 reference level. CFI = comparative fit index; TLI = Tucker-Lewis index; RMSEA = root mean square error of approximation; SRMR = standardized root mean square residual.

Table S4

Prediction Equation Accuracy: In-Sample and Leave-One-Scenario-Out Cross-Validation

Specification	Index	Quantile	In-sample MAE	In-sample MdAE	In-sample Max AE	LOOCV MAE	LOOCV MdAE	LOOCV Max AE	N scenarios
Panel A. Main specification: $\log_{10}(N)$	CFI	$\tau = .05$	0.0287	0.0204	0.0720	0.0473	0.0528	0.0949	36
	TLI	$\tau = .05$	0.0350	0.0249	0.0879	0.0578	0.0645	0.1158	36
	RMSEA	$\tau = .95$	0.0046	0.0036	0.0127	0.0055	0.0047	0.0131	36
	SRMR	$\tau = .95$	0.0049	0.0035	0.0159	0.0065	0.0051	0.0182	36
Panel B. Robustness specification: categorical N	CFI	$\tau = .05$	0.0195	0.0110	0.0765	0.0229	0.0187	0.0771	36
	TLI	$\tau = .05$	0.0238	0.0134	0.0935	0.0280	0.0228	0.0941	36
	RMSEA	$\tau = .95$	0.0037	0.0033	0.0112	0.0051	0.0048	0.0115	36
	SRMR	$\tau = .95$	0.0038	0.0030	0.0139	0.0051	0.0041	0.0155	36

Note. Accuracy was computed for quantile-regression prediction equations fitted to scenario-level cutoffs calibrated at $\alpha = .05$. Absolute error is the absolute difference between the observed scenario-level cutoff and the predicted cutoff. The main specification used $\log_{10}(N)$; the robustness specification treated N as a three-level categorical predictor. LOOCV left out one Estimator × Category Handling × Sample Size × Loading Regime scenario at a time. CFI and TLI models used $\tau = .05$; RMSEA and SRMR models used $\tau = .95$. MAE = mean absolute error; MdAE = median absolute error; Max AE = maximum absolute error; LOOCV = leave-one-scenario-out cross-validation; CFI = comparative fit index; TLI = Tucker-Lewis index; RMSEA = root mean square error of approximation; SRMR = standardized root mean square residual.

Figure S1

Estimator-Specific Scenario-Calibrated Correct-Model Cutoffs Within Each Scenario

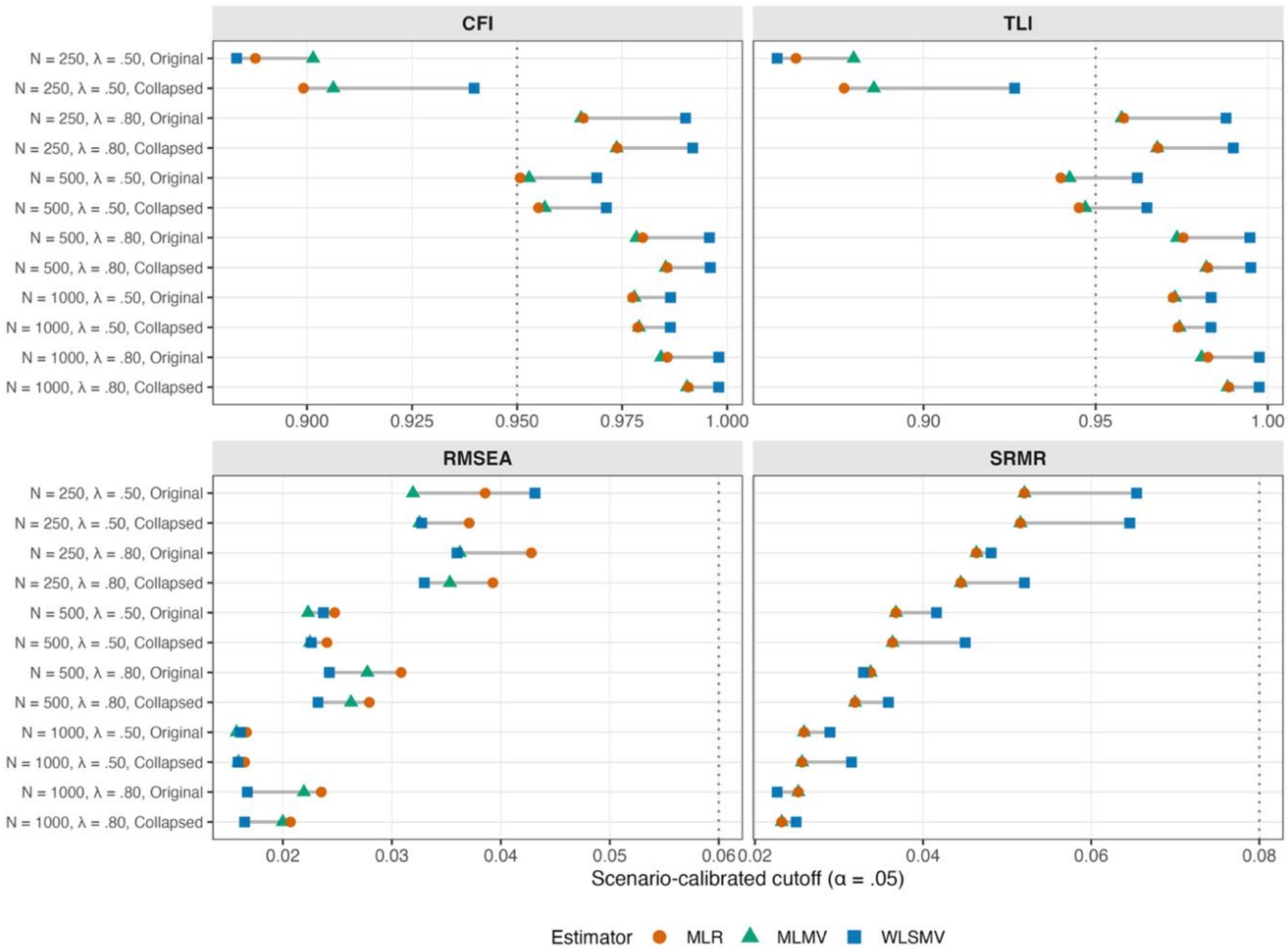

Note. Estimator-specific scenario-calibrated Correct-model cutoffs within each sample-size, loading-strength, and category-handling scenario. Points show scenario-level cutoffs calibrated at $\alpha = .05$ from the Correct-model reference distributions for each estimator. Gray segments span the minimum to maximum cutoff across estimators within each scenario. Dotted vertical lines mark conventional heuristic benchmarks: CFI/TLI = .95, RMSEA = .06, and SRMR = .08. x-axis scales vary across fit indices. Original indicates that the sparse five-category response scale was retained. Collapsed indicates that sparse categories were collapsed according to the deterministic recoding rules before model estimation. CFI = comparative fit index; TLI = Tucker-Lewis index; RMSEA = root mean square error of approximation; SRMR = standardized root mean square residual.
